# Distribution of iron- and sulfate-reducing bacteria across a coastal acid sulfate soil (CASS) environment: implications for passive bioremediation by tidal inundation

**DOI:** 10.3389/fmicb.2015.00624

**Published:** 2015-07-03

**Authors:** Yu-Chen Ling, Richard Bush, Kliti Grice, Svenja Tulipani, Lyndon Berwick, John W. Moreau

**Affiliations:** ^1^School of Earth Sciences, University of MelbourneMelbourne, VIC, Australia; ^2^Southern Cross GeoScience, Southern Cross UniversityLismore, NSW, Australia; ^3^Department of Chemistry, Western Australia Organic and Isotope Geochemistry Centre, The Institute for Geoscience Research, Curtin UniversityPerth, WA, Australia

**Keywords:** acid sulfate, microbial community, iron-reducing, sulfate-reducing, iron sulfides, geomicrobiology, wetlands, remediation

## Abstract

Coastal acid sulfate soils (CASS) constitute a serious and global environmental problem. Oxidation of iron sulfide minerals exposed to air generates sulfuric acid with consequently negative impacts on coastal and estuarine ecosystems. Tidal inundation represents one current treatment strategy for CASS, with the aim of neutralizing acidity by triggering microbial iron- and sulfate-reduction and inducing the precipitation of iron-sulfides. Although well-known functional guilds of bacteria drive these processes, their distributions within CASS environments, as well as their relationships to tidal cycling and the availability of nutrients and electron acceptors, are poorly understood. These factors will determine the long-term efficacy of “passive” CASS remediation strategies. Here we studied microbial community structure and functional guild distribution in sediment cores obtained from 10 depths ranging from 0 to 20 cm in three sites located in the supra-, inter- and sub-tidal segments, respectively, of a CASS-affected salt marsh (East Trinity, Cairns, Australia). Whole community 16S rRNA gene diversity within each site was assessed by 454 pyrotag sequencing and bioinformatic analyses in the context of local hydrological, geochemical, and lithological factors. The results illustrate spatial overlap, or close association, of iron-, and sulfate-reducing bacteria (SRB) in an environment rich in organic matter and controlled by parameters such as acidity, redox potential, degree of water saturation, and mineralization. The observed spatial distribution implies the need for empirical understanding of the timing, relative to tidal cycling, of various terminal electron-accepting processes that control acid generation and biogeochemical iron and sulfur cycling.

## Introduction

Coastal acid sulfate soils (CASS) constitute a major global environmental problem (Dent and Pons, 1995; White et al., 2007; Ljung et al., 2009). The resulting problems include fish kills (Powell and Martens, 2005; Stephens and Ingram, 2006), decreased rice yields (Bronswijk et al., 1995), release of greenhouse gases such as methane and dinitrogen oxide (Denmead et al., 2007), sulfur dioxide emissions (Macdonald et al., 2004), construction damage (Crammond, 2002), and changed mobility of toxic metals (Burton et al., 2008). In Australia, roughly $10 billion worth of acid sulfate soil “legacy” impacts remain (Fitzpatrick, 2003), and Australia contains only about 18% of acid sulfate soils worldwide (Ljung et al., 2009).

Although some CASS environments result from tectonic uplift (Åström and Björklund, 1995), anthropogenic drainage of wetlands generally accounts for most recent and modern CASS formation (Rosicky et al., 2004; Ljung et al., 2009). Drainage allows oxygen to penetrate further into the subsurface, resulting in oxidation of iron sulfides and release of protons and sulfuric acid. For instance, the oxidation of pyrite by molecular oxygen (Hicks et al., 1999):

(1) $$FeS_{2}\;+\;3.75O_{2}\;+\;3.5H_{2}O\to Fe{\left(OH\right)}_{3}\;+\;4H^{+}\;+\;2SO_{4}^{2-}$$

releases 4 moles of acid and 2 moles of sulfate per mole of pyrite. Microbial intervention will expedite the reaction rate by co-oxidation of ferrous iron, with the product Fe^3+^ acting as a strong oxidant of pyrite.

Low pH pore waters promote the mobility of toxic heavy metals such as aluminum and manganese (Willett et al., 1992; Sammut et al., 1996), which can be partitioned into metal-sulfides (Moreau et al., 2013). A pH decrease from 3.7 to 1.9 can result in a dissolved aluminum increase from 0.9 to 40 M (Van Breemen, 1973). Aluminum hydrolysis generates 3 moles of acid from one mole of aluminum and decreases pH further (Hicks et al., 1999):

(2) $$Al^{3+}\;+\;H_{2}O\to Al{\left(OH\right)}^{2+}\;+\;H^{+}$$

(3) $$Al{\left(OH\right)}^{2+}\;+\;H_{2}O\to Al{\left(OH\right)}_{2}^{+}\;+\;H^{+}$$

(4) $$Al{\left(OH\right)}_{2}^{+}\;+\;H_{2}O\to Al{\left(OH\right)}_{3}\;+\;H^{+}$$

The most common treatments for CASS-related contamination are lime neutralization and seawater inundation (White et al., 1997; Johnston et al., 2009a,b). The primary disadvantage of lime treatment is the relatively high cost and need for extensive and continued maintenance. In the Great Barrier Reef catchments in Australia, it was estimated that $62 million AUD would be needed for lime treatment over 6.7 × 10^5^ hectares of CASS-affected regions (Powell and Martens, 2005). However, lime addition accounts for less than 1% of the alkalinity in a lime-assisted tidal inundation treatment (Johnston et al., 2012). Re-flooding by seawater is hence a more cost effective treatment strategy, as marine bicarbonate accounts for 25~40% of total alkalinity generation (Johnston et al., 2012). The processes of microbial sulfate and iron reduction triggered by seawater flooding contribute to more than 50% of total alkalinity (Johnston et al., 2012). Both iron and sulfate reduction consume protons:

(5) $$Fe{\left(OH\right)}_{3}+0.25CH_{2}O+2H^{+}\to Fe^{2+}+0.25CO_{2}+2.75H_{2}O$$

(6) $$SO_{4}^{2-}+2(CH_{2}O)\to 2HCO_{3}^{-}+H_{2}S$$

and can lead to formation of mackinawite (FeS) or pyrite (FeS_2_), both of which can promote the immobilization of arsenic, a common toxic metalloid in CASS environments (Burton et al., 2011).

Microorganisms can also influence other biogeochemical cycles in natural and contaminated wetlands. However, little research has been conducted to provide detailed information about microbial processes and bioremediation efficiency specifically in CASS systems. Previous microbiological studies of CASS-like systems have discussed the vertical depth-distribution of sulfur- and iron-oxidizing bacteria in a paddy field (Ohba and Owa, 2005), revealed high microbial biomass and activity at the Baltic coast, Finland (Simek et al., 2013) and compared microbial communities from Ostrobothnian, Finland, with those found in acid mine drainage (AMD) (Wu et al., 2013). To understand the potential for microbial acid generation and biogeochemical cycling in CASS-impacted sediments, a comprehensive, spatially integrative resolution of microbial “functional guild” distribution is required. Specifically, we need to know more about the distribution and diversity of sulfur- and iron-cycling microorganisms. Previous studies have long established that iron reducing bacteria (IRB) can out-compete sulfate reducing bacteria (SRB) for limited electron donor when the environment is non-limiting in ferric iron (e.g., Lovley and Phillips, 1987; Chapelle and Lovley, 1992). However, we hypothesized that increases in the concentrations of organics in CASS systems decrease competition between IRB and SRB by increasing thermodynamic energy availability relative to enzyme kinetics. We present results and analyses from an investigation of whole community 16S rRNA genes amplified from CASS-impacted sediments from the East Trinity wetlands (Cairns, Australia), a tidally influenced wetland located on the northeast coast of Australia. Gene data are interpreted in the context of environmental and organic geochemical data acquired from the site. The results of this study were analyzed in the context of soil type and sediment lithology, degree of pore water saturation, tidal inundation and turbidity, acidity and organic carbon availability to understand the factors that shape microbial community structure and activity.

## Materials and methods

### Field site and soil sampling

The majority of CASS environments were formed during the last Holocene post-glacial period, as rising sea levels promoted the deposition of iron sulfide minerals (Dent, 1986). The East Trinity wetlands study site is characterized by abundant potential CASS in Holocene soil layers that formed when high rates of organic matter degradation under warm temperatures stimulated iron and sulfate reduction (White et al., 1997). In the 1970s, large-scale drainage of seawater exposed CASS to air and resulting in soil acidification and heavy metal contamination problems in this area and nearby ecosystems. Since 2001, tidal exchange remediation is being practiced in this area (QASSIT, 2000).

**Figure 1:**
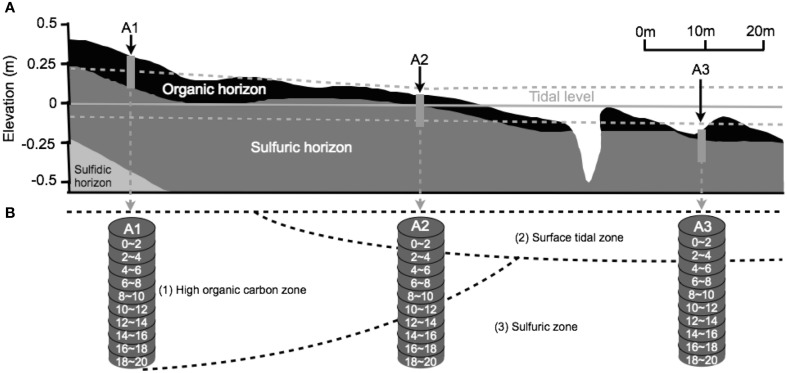
**The distribution of soil layers, water saturation state, and β diversity. (A)** The *in situ* soil layers, water saturation state, and sampling sites. **(B)** Three zones at East Trinity site divided by β diversity. Figures are modified from Burton et al. (2011) and Johnston et al. (2011a,b).

The pH and Eh values of each core section were measured in the field with a portable pH/Eh meter by Thermo Scientific Orion 5-Star Portable Plus pH/ORP/ISE/Conductivity/DO meter with a Model 9678BN Pt-Ag/AgCl combination electrode. ORP measurements were calibrated to a standard hydrogen electrode at 20°C. The resolution and relative accuracy of pH and ORP are 0.01 and ±0.002, and 0.1 mV and ±0.2, or 0.05%, respectively. Soil samples were collected from a sub-catchment of Firewood Creek in the East Trinity Wetland (145°80'E, 16°94'S), northeast Australia (Burton et al., 2011; Johnston et al., 2012). Three 20 cm-deep sediment cores were collected along an upland to seawater transect (Figure 1, sampling sites A1 to A3, respectively) during a low tide period, and then sectioned into 2 cm intervals. Sediments were collected into 15 mL sterile bottles containing 3 mL of RNA*later*™ RNA stabilization reagent and then preserved at −80°C in a freezer until DNA extractions were performed. Chemical data were measured in the field by insertion of the electrode into soil/pore water at each of the sampling depths, with several rinses with nanopure water in between measurements. The sampling transects incorporated both the surface, or O horizon consisting of surficial organic deposits (Johnston et al., 2011b, 2012), and sulfuric horizon, consisting of actual acid sulfate soils characterized by low soil pH (Hicks et al., 1999).

### Organic geochemistry analyses

Organic geochemical analyses were performed on aliquots of the top 6 cm of sediment cores from the transect (A1–A3) and two control sites located (i) outside of the tidal bund-wall (not affected by drainage and CASS formation; “Mangrove Site”) and (ii) at an CASS site not treated with tidal inundation (“Acidic Site”). The samples were freeze-dried, homogenized and Soxhlet-extracted (48 h) in a mixture of dichloromethane (90%) and methanol (10%). Elemental sulfur was removed with activated copper pellets. The extracts were separated into three fractions (aliphatics, aromatic and polar) by silica gel column chromatography using eluents of increasing polarity (e.g., Nabbefeld et al., 2010). Aliquots of the dried polar fractions were derivatised in a 3:2 mixture of bis(trimethylsilyl)-trifluoroacetamide (BSTFA) and anhydrous pyridine for (20 min) at 60–70°C and analyzed using GC-MS within a few hours.

Gas chromatography-mass spectrometry (GC-MS) analyses of aromatic and derivatised polar fractions were performed on an Agilent 6890/5973 GC-MS equipped with an Agilent 6890 autosampler and a 60 m × 0.25 mm i.d. × 0.25 μm film thickness DB5-MS column (J&W Scientific). Aromatic fractions were injected in splitless mode. Polar fractions were injected in splitless cool on column mode into an Alltech pre-column (2 m x 0.53 mm i.d.) fitted to the DB5-MS column. Helium was used as a carrier gas at a constant flow of 1.1 and 1.2 mL/min for aromatic and polar fractions, respectively. The GC oven was heated from 40 to 325°C at 3°C/min with initial and final hold times of 1 and 30 min for aromatic fractions and from 50 to 320°C at 6°C/min with initial and final hold times of 1 and 20 min for polar fractions. The MS was operated at 70 *eV* and acquired full scan mass spectra (50–550 Daltons and 50–750 Daltons for aromatic and polar fractions, respectively) at ~3 scans/s and a source temperature of 230°C. Peak assignments were based on correlation of GC retention time and mass spectral data with reference compounds, library spectra or other published data.

### DNA extraction and 454 pyrosequencing

Each DNA extraction used ~0.3 g of sediments (wet weight) with the PowerSoil DNA Isolation Kit (Mo Bio Laboratories, Inc. Carlsbad, CA) according to the manufacturer's protocol. DNA samples were sent for 454 pyrosequencing at the Australian Centre for Ecogenomics (ACE; Brisbane, Australia). A first round of PCR was conducted with SSU803F (combinations of 803Fa 5′-TTAAGATACCCTGGTAGTC-3′, 803Fb 5′-TTAGATACCCSGGTAGTC-3′, 803Fc 5′-TTAGATACCCTHGTAGTC-3′, 803Fd 5′-TTAGATACCCTGGTAGTC-3′ in a ratio of 2:1:1:1, *E. coli* position 2305–2322, Brosius et al., 1981) and SSU1392R (5′-ACG GGC GGT GWG TRC-3′, *E. coli* position 2908–2923, Brosius et al., 1981) primers used in the Fisher kit, with 1U Taq, dNTP at a final concentration of 0.2 mM, primers at a final concentration of 0.2 μM, MgCl_2_ at a final concentration of 2 mM and BSA at a final concentration of 0.3 mg/mL. Thermal cycling had an annealing temperature of 55°C with 30 cycles. ACE has done extensive testing with this protocol and biases in amplication have been minimized. Then 2 μL of the first PCR product was used for a second PCR with no clean up to add barcodes (Multiplex Identifiers, MIDs), using the same reagents and conditions as for the first PCR but for 10 cycles. The PCR products were then quantified on the TapeStation and pooled at equal concentrations. The pooled DNA was gel extracted and amplified by emulsion PCR for sequencing. The Roche 454 sequencing (GS FLX Titanium chemistry) was performed with Roche 454 protocols.

### 16s rRNA gene sequences analyses

DNA sequences were analyzed using the software environment Mothur (Schloss et al., 2009) v.1.32.1 following the Mothur 454 SOP (accessing date: Dec 2013) (Schloss et al., 2011). Raw sequence data were deposited to the Sequence Read Archive (SRA) of NCBI under the accession number: PRJNA275357. Sequences were removed for which the average quality dropped below 35. This step removed 7605 of a total 169,237 reads. Unique sequences were identified and the closest reference sequences were selected from SILVA bacterial and archaeal databases by the kmer search method, followed by a needleman alignment to make pairwise alignments between reference and candidate sequences (Schloss, 2010). Aligned sequences were checked to keep the most overlapping positions. Alignment results showed that 97.5% sequences had same ending position; thus we eliminated the 2.5% of sequences that ended before this position. Start positions were optimized to 85% sequence position equivalency, and sequences which started later were removed. Finally, columns in the alignments were filtered. The remaining 136,463 sequences were 206 bp in average length. Sequences were pre-clustered and 2052 reads were detected and removed as pyrosequencing errors and chimeras by the uchime program (Edgar et al., 2011). Taxonomy information was assigned to sequences with a cutoff of 50% (Claesson et al., 2009) by Naïve Bayesian classifier (Wang et al., 2007), with Ribosomal Database Project (RDP) references. Sequences with similarities higher than 97% were assigned to one OTU (operational taxonomic unit).

Samples were randomly resampled to 1498 reads for different calculations. The Good's coverage was calculated, which represents the ratio of OTUs that have been sampled once to the total number of sequences. The Chao 1 index was determined to estimate the richness of a sample based on the numbers of observed OTUs, singletons and doubletons. The inverse Simpson and Shannon indices were calculated to represent OTU diversity (alpha diversity) for each sample. The evenness values were used to evaluate the distribution evenness of relative OTU abundances. The beta diversity, which represents differentiation among each sample, was calculated and represented in two ways: a dendrogram which was calculated using the Jaccard index, then clustered using the UPGMA algorithm; and a principle coordinate analysis (PCoA) calculated using the Yue and Clayton index (Yue and Clayton, 2005). An ANOVA test was used to evaluate variability in diversity across sites.

### Kinetic drive evaluation

A combined thermodynamic-kinetic rate law was used to evaluate factors controlling microbial metabolic rates in the CASS system (Jin and Bethke, 2003, 2005):

(7) $$v=\text{k}\left[X\right]\;F_{T}\;F_{K}$$

Metabolic rate *v* is the product of the rate constant *k*, microbial biomass concentration [*X*], thermodynamic factor *F_T_* and the kinetic factor *F_K_*. The range of values for kinetic and thermodynamic factors lies between 1 and 0. Larger factors (toward 1) represent less kinetic or thermodynamic limitations, or that the reaction is far from equilibrium and the forward direction overwhelms the reverse direction. If the reaction is close to equilibrium, the factors decrease toward to 0, which means little energy is available for microorganisms. This rate law is built on Monod and Michaelis-Menten kinetics (Monod, 1949; Michaelis et al., 2011), and is extended to consider the reverse reactions and include a thermodynamic potential factor. The consideration of reverse reactions and thermodynamic factors can be neglected where the environments contain abundant energy such that the forward reaction overcomes the reverse direction (Jin and Bethke, 2007). Thus, the model can be used to test the assumption that organic carbon substrates are non-limiting in the CASS environment under study.

Acetate is the most common organic substrate available for sulfate reduction in many environments (Lovley and Klug, 1982):

(8) $${\text{SO}}_{4}^{2-}\;+\;{\text{CH}}_{3}{\text{COO}}^{-}↔{\text{HS}}^{-}\;+\;{2\text{HCO}}_{3}^{-}$$

The thermodynamic factor *F_T_* of acetotrophic sulfate reduction is:

(9) $$F_{T}=\;1-\text{ exp}\left(\frac{△G_{A}+△G_{C}}{\chi RT}\right)$$

where *R* and *T* are the gas constant (8.314 J/Kmol) and absolute temperature (298 K was used in this study), χ is average stoichiometric number with a suggested value of 6 used in this study (Jin et al., 2013). Δ*G_C_* is the energy conserved by SRB per mole of sulfate, which is estimated to be 33–47 kJ/mol (Jin and Bethke, 2009) with a value of 45 used in this study (Jin et al., 2013). The energy available in the environment Δ*G_A_* is the Gibbs free energy of reaction (Equation 8):

(10) $$△G_{A}=△G_{T}^{0}\;+\;RT\ln\frac{\gamma_{{\text{HS}}^{-}}\left[{\text{HS}}^{-}\right]\;\cdot \;\gamma_{{\text{HCO}}_{3}^{-}}{\left[{\text{HCO}}_{3}^{-}\right]}^{2}}{\gamma_{{\text{SO}}_{4}^{2-}}\left[{\text{SO}}_{4}^{2-}\right]\;\cdot \;\gamma_{{\text{CH}}_{3}{\text{COO}}^{-}}\left[{\text{CH}}_{3}{\text{COO}}^{-}\right]}$$

where γ_*i*_ is the activity coefficient, [i] is the concentration of reactant or product i, and the Δ*G*^0^_*T*_ is the standard Gibbs free energy at absolute temperature, T°K. The activity coefficient γ_*i*_ used in this study was derived using the Geochemist's Workbench software (Bethke, 2007). The activity coefficient ranges for SO^2−^_4_, CH_3_COO^−^, HS^−^, and HCO^−^_3_ are 0.1561–0.16508, 0.6825–0.6842, 0.6395–0.6426, and 0.6825–0.6842, respectively. The value of Δ*G*^0^_*T*_ is −47.6 (kJ/mole) for acetotrophic sulfate reduction (Thauer et al., 1977; Sawadogo et al., 2013). The SO^2−^_4_ and CH_3_COO^−^ concentration profiles were modified from previous research at the same study site (**Figure 8**, Supplementary Table 2), the sulfate concentration range was 3–45 mM, the acetate concentration range was 0–95 mM, the sulfide value used the theoretically highest amount 2 μM since the sulfide concentration is below the detection limit (Supplementary Table 2) (Burton et al., 2011), and the bicarbonate concentrations used the highest values measured in the study site 1.6 mM (Johnston et al., 2011b). Other chemical concentrations used data reported from the same study site (Supplementary Table 2) (Ward et al., 2014).

The kinetic factor, *F_K_*, derived from Monod (Monod, 1949) and Michaelis-Menten kinetic equations:

(11) $$v=v_{max}\frac{\left[{\text{SO}}_{4}^{2-}\right]}{K_{{\text{SO}}_{4}^{2-}}+\left[{\text{SO}}_{4}^{2-}\right]}\frac{\left[{\text{CH}}_{3}{\text{COO}}^{-}\right]}{K_{{\text{CH}}_{3}{\text{COO}}^{-}}+\left[{\text{CH}}_{3}{\text{COO}}^{-}\right]}$$

where *v_max_* is the maximum metabolic rate, and the kinetic factor is described as:

(12) $$F_{K}=\;\frac{\left[{\text{SO}}_{4}^{2-}\right]}{K_{{\text{SO}}_{4}^{2-}}+\left[{\text{SO}}_{4}^{2-}\right]}\frac{\left[{\text{CH}}_{3}{\text{COO}}^{-}\right]}{K_{{\text{CH}}_{3}{\text{COO}}^{-}}+\left[{\text{CH}}_{3}{\text{COO}}^{-}\right]}$$

where *K* is the half-saturation constant; this study used values of 5.0 × 10^−6^ M and 9.17 × 10^−4^ M as *K*_CH_3_COO^−^_ and *K*_SO^2−^_4__, respectively (Jin et al., 2013).

### Plotting software

The software R (Statistical Package, 2009), R package gplots (Warnes et al., 2009), Microsoft Excel, iWork Numbers, and FigTree were used for generating plots.

## Results

### *In situ* geochemical measurements

The pH of sediments generally decreased along the transect from the sea toward the upland site (“A1”) for the upper portions (0–10 cm) of each core, with values from 3.29 to 6.13 at site A1, 4.38 to 5.92 at site A2, and 6.08 to 6.43 at site A3. In contrast, pH values were similar for all three sites for the lower portions (10–20 cm), ranging from 5.97 to 6.79. The Eh values decreased with depth within each core (from 51 to -127 mV at site A1, 20 to −207 mV at site A2, and 10 to −459 mV at site A3), but generally increased along the transect from the sea toward the upland site at each depth.

### Organic geochemistry analyses

**Figure 2:**
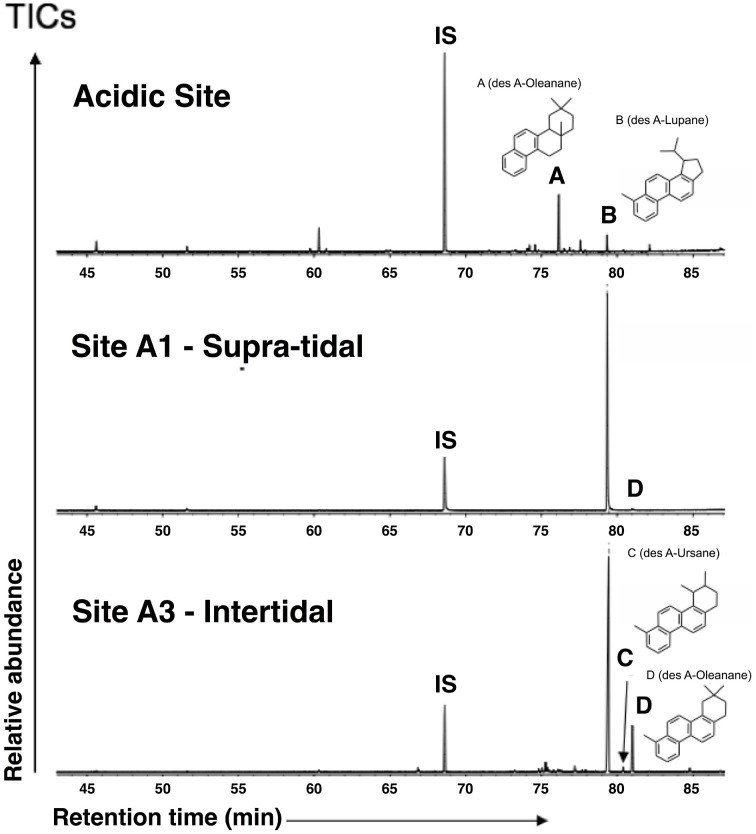
**Total ion chromatograms from GC-MS analyses of aromatic fractions from surface sediments (study sites A1, A3, and an acidic control site not treated with tidal inundation)**. Aromatic fractions from the study sites are dominated by a des A-Lupane whereas the aromatic fraction from the “Acidic Site” was dominated by a des A-Oleanane. IS, internal standard (c.f. Eiserbeck et al., 2012 and references therein).

The polar fractions from surface sediments of each transect site, as well as from the acidic control site, were dominated by a suite of plant-derived pentacyclic 3-oxy triterpenoids, including oleanolic, betulinic and ursolic acids, which showed a similar distribution in all samples. However, these compounds were absent in the polar fractions obtained from the mangrove control site, which was dominated by the triterpenoid taraxerol. In the aromatic fractions, pentacyclic triterpenoid derivatives were highly abundant (Figure 2). However, the aromatic fraction from the “Acidic Site” was dominated by a des-A-oleanane, whereas in the transect sites a des-A-lupane was the most abundant compound.

### DNA sequences analyses

**Figure 3:**
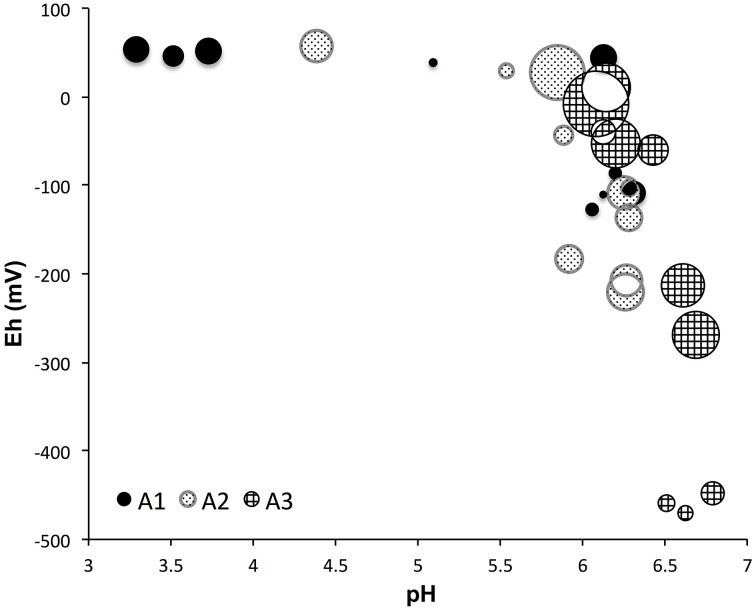
**The alpha diversity (inverse Simpson) vs. pH and Eh values among three sampling sites, the diversity values are proportional to the area of bubbles**. The more positive Eh values indicate more oxidized environments. Diversity showed significant difference among three sites (*P* < 0.01, ANOVA).

The 95% confidence intervals of alpha diversity and richness were computed and the higher and lower boundaries were checked (Supplementary Table 2) to make sure the variations among samples were greater than the 95% confidence interval limitations. Average sampling coverage values showed significant increase along the transect moving away from the sea (72% for site A3, 81% for site A2, and 88% for site A1, *P* < 0.001, ANOVA). Chao richness estimates, which estimate the number of phylogenetically different OTUs (3% or more different in sequence composition), displayed a significant decrease from site A3 to A1 (*P* < 0.001, ANOVA), with the highest values in the upper part of site A3 (3668 reads), followed by sites A2 (1707 reads) and A1 (1128 reads), respectively. Both Shannon and Simpson diversity indices showed a significant difference among the three sites (*P* < 0.01, ANOVA), which is shown on the pH and Eh diagram with the inverse Simpson index (Figure 3). The inverse Simpson index was in the range of 6–500 and Shannon index was in the range of 2.3–6.4 (Supplementary Table 2). Simpson's evenness indices were all below 0.37, which indicates a relatively even microbial distribution.

**Figure 4:**
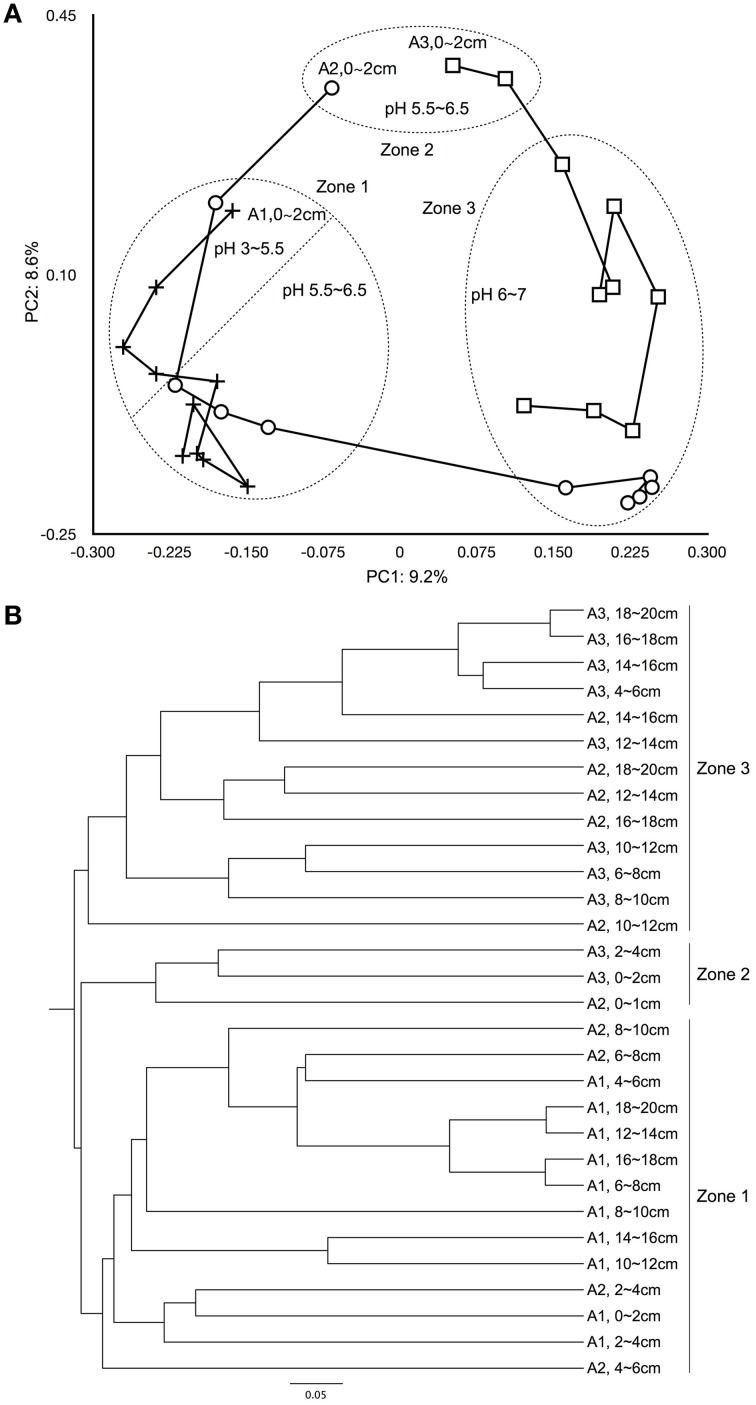
**Beta diversity analysis comparing microbial structure similarity among samples. (A)** Principal coordination analysis among samples from the Jaccard calculator (PC1 = 9.2%, PC2 = 8.6%). The “+” represents samples from site A1, the “○” represents samples from site A2, and the “□” represents samples from site A3. **(B)** Yue and Clayton measure of samples similarity.

**Figure 5:**
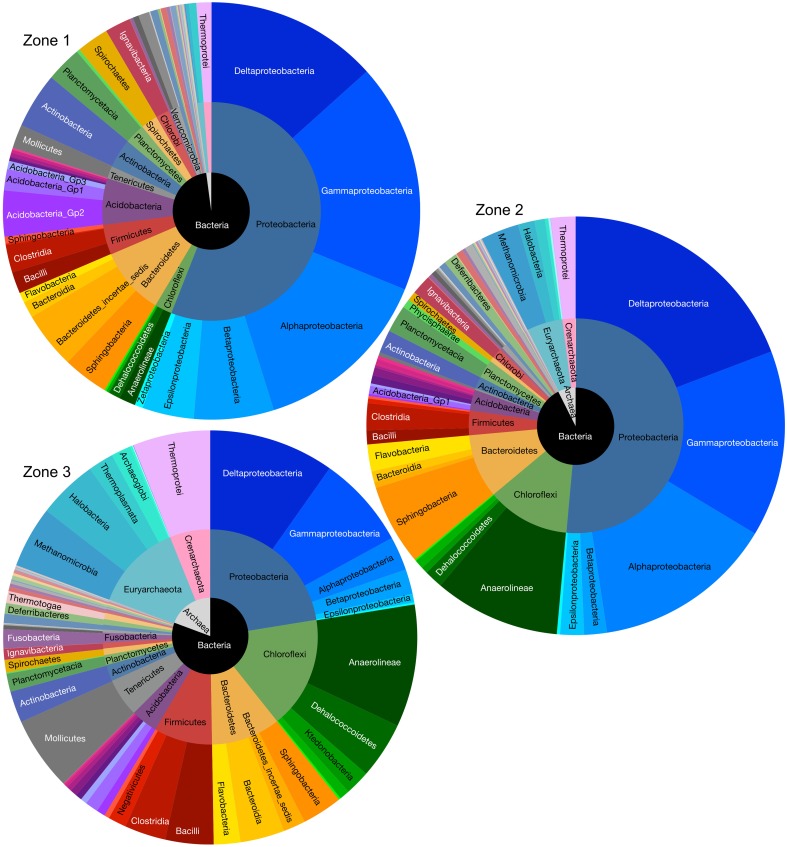
**Microbial abundance and compositions of Zone 1, Zone 2, and Zone 3 in kingdom (inner circle), phylum (middle circle), and class (outer lever) taxonomic levels**.

Beta diversity analysis illustrated the degree of similarity in microbial composition for each site and sample (Figure 4). Sequences tended to group into clusters consistent with major physico-chemical changes in soil profile, as well as with variations in the degree of soil moisture saturation (Figure 1). Relative percentage representations of microbial community structure, separated into domain, phylum and class for each zone are shown in Figure 5. The inner, middle and outer circles represent domain, phylum and class levels, respectively (Figure 5).

Bacteria comprised ~89% and archaea ~11% of the prokaryotic community structure, taken across all sites within the East Trinity wetlands. In total 30 bacterial and three archaeal phyla (*nanoarchaeota* occupied only 0.04% and therefore did not show observable area in Figure 5) were identified. *Proteobacteria* was the most abundant phylum detected at any site, contributing ~39% of the total 16S rRNA gene sequences. The nine most abundant phyla recovered accounted for ~93% of these sequences. The relative abundance of each shown class or phylum differed between zonations. For example, the abundance of *deltaproteobacteria* had the highest abundance at Zone 2 (19%, compared to 13% at Zone 1 and 10% at Zone 3). The *gamma*-, *beta*-, and *alpha-proteobacteria* exhibited higher sequence abundances at Zone 1 (38% totally, compared to 30% at site Zone 2 and 12% at site Zone 3). The *Chloroflexi*, *Bacteroidetes*, *Firmicutes, Euryarcheota*, and *Crenarchaeota* showed greater abundances in Zone 2 (22%) and Zone 3 (39%), compared to Zone 1 (8%).

**Figure 6:**
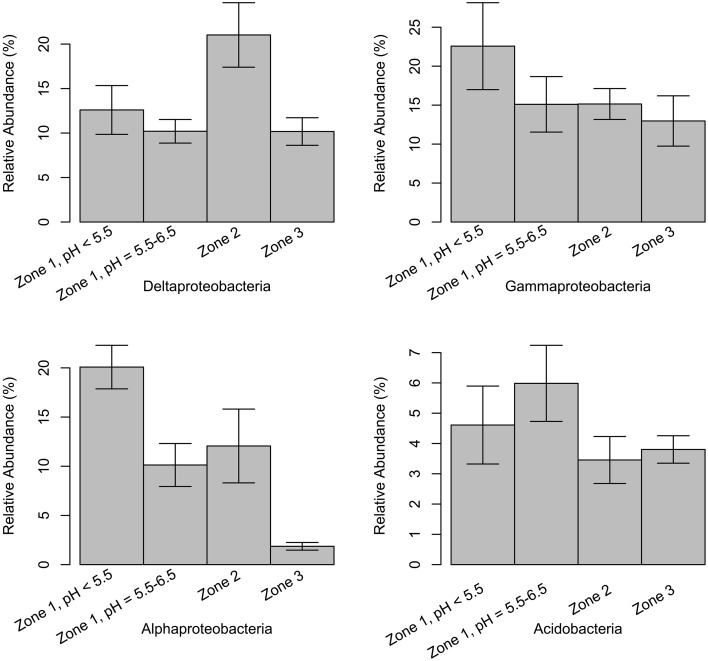
**Relative abundance of classes *delta-, gamma-, alpha-proteobacteria*, and *Acidobacteria* from Zone 1 to Zone 3**. Samples in Zone 1 are separated into two parts based on the pH values and structure similarity (Figure 3A). The error bars show the 95% higher and lower confidence intervals around mean values.

**Figure 7:**
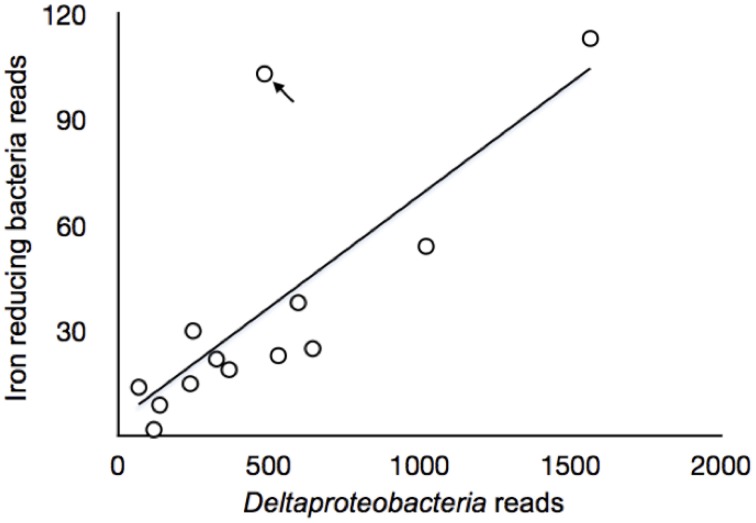
**Correlations between sequence reads of samples collected of sulfate-reducing *deltaproteobacteria* and iron reducing bacteria in Zone 3**. The arrow points to the A3 4–6 cm, where the sample differed from all other samples and was discussed in the text.

**Figure 8:**
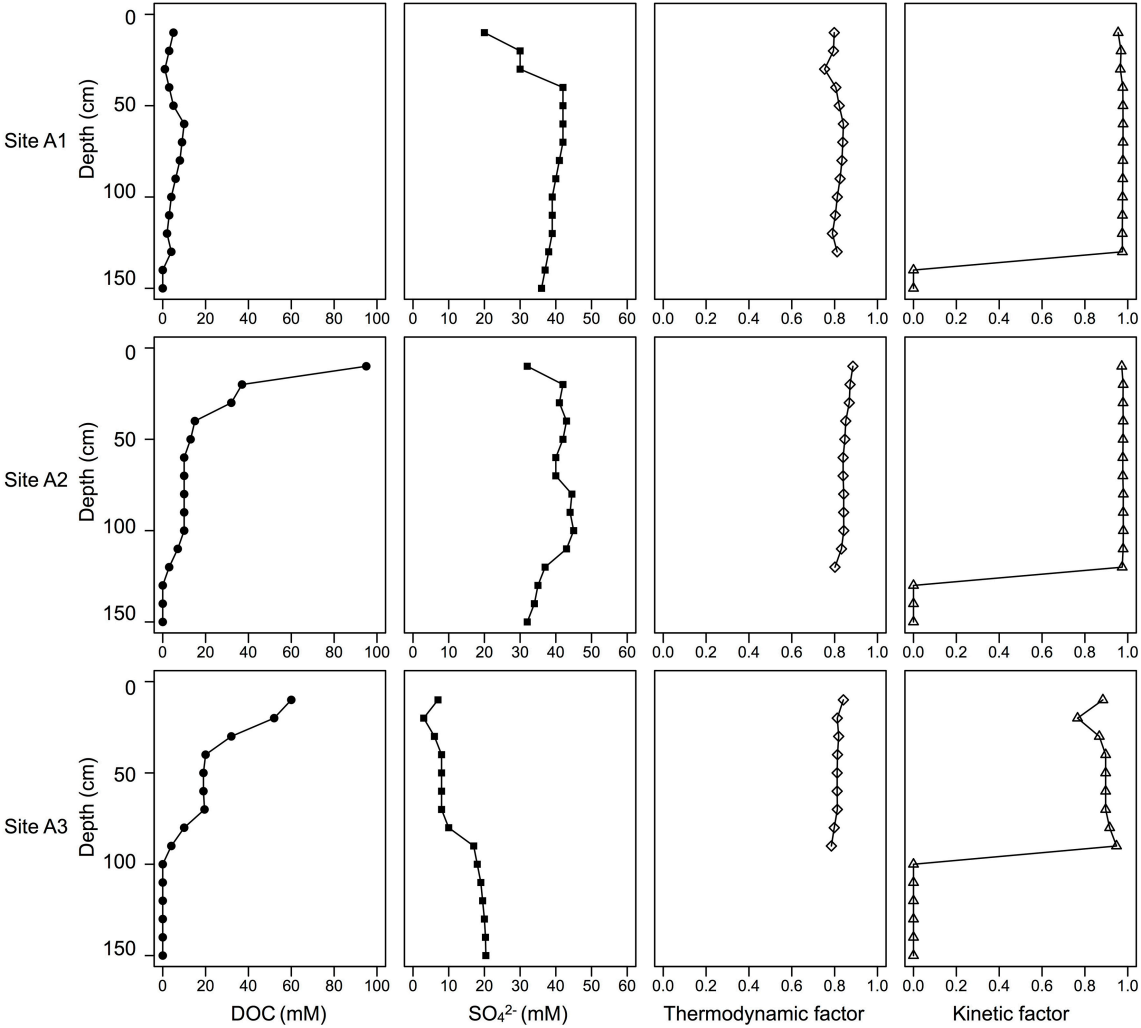
**Depth profile of DOC, SO^2−^_4_, labile Fe(III), crystalline Fe(III), soil density, and kinetic drives of heterotrophic sulfate and iron reductions**. The values of DOC, SO^2−^_4_, labile Fe(III), crystalline Fe(III), and soil density are modified from Burton et al. (2011).

Classes *delta*-, *gamma*-, *alpha-proteobacteria*, and *Acidobacteria* were selected to compare their relative abundances among different zonations and other environments, such as marine sediments and AMD systems (Figure 6). Several genera, which have been reported by previous researchers to have iron reducing ability, were picked from this study to represent the abundance of IRB (Table 1). The iron-reducing bacterial reads were proportional to those of *deltaproteobacteria*, and iron-oxidizing bacterial reads were proportional to those of sulfur-oxidizing bacteria, in the organic and sulfuric horizons (Figure 7). The kinetic drives of heterotrophic sulfate and iron reductions were calculated to be close to unity in the study area (top 20 cm, Figure 8 and Supplementary Table 3), which supports the lack of kinetic inhibition for metabolisms in this CASS system.

**Table 1:** **Genera of iron-reducing bacteria**.

**Genus name**	**References**
**IRON-REDUCING**	
*Paraferrimonas*	Khan and Harayama, 2007
*Aciditerrimonas*	Itoh et al., 2011
*Desulfuromonas*	Coates et al., 1995
*Bacillus*	Pollock et al., 2007
*Pelobacter*	Lovley et al., 1995
*Desulfuromusa*	Vandieken, 2006
*Desulfitobacterium*	Finneran et al., 2002
*Thiobacillus*	Temple and Colmer, 1951
*Geobacter*	Lovley et al., 1993; Caccavo et al., 1994
*Desulfosporosinus*	Robertson et al., 2001
*Ferroplasma*	Golyshina et al., 2000
*Geothrix*	Coates et al., 1999
*Shewanella*	Roh et al., 2006
*Ferribacterium*	Cummings et al., 1999
*Ferrimonas*	Rosselló-Mora et al., 1995

## Discussion

### Organic source and preservation

There is more than 20% (by weight) organic carbon in the organic horizon at East Trinity wetlands (Hicks et al., 1999). This relatively high organic matter content most likely results from a mode of origin and preservation uniquely associated with re-flooded CASS environments. Mangroves can slow surface water flow rates and reduce wave scour, which favors fine particle trapping and organic matter accumulation (Young and Harvey, 1996; Alongi, 2008). Sediments in mangrove swamps usually contain a large amount of organic matter (Kristensen et al., 2008). Mineralogy may play a role in preserving organic carbon in CASS systems; the oxidation of iron sulfides generates secondary iron minerals such as ferrihydrite and goethite in the study site (Hicks et al., 1999; Johnston et al., 2010), which have been shown to preserve mineral-bound organic carbon in subsoils (Kogel-Knabner et al., 2008). Environmental factors including salinity, soil pH or the tidal-inundation level at the sites control primary production; in particular the plant type will shape the types of organics available for microbial degradation.

Changes in plant types due to tidal inundation treatment contributed a large amount of organic matter input, while organo-mineral interactions resulted in unusual preservation of organic acids. Combined with invertebrate decay in the mangrove area, these factors contributed to the high abundance of organic matter in the system, and hence influenced microbial structures and distributions. In our wetland site, *Melaleuca* trees (mostly *Melaleuca leucadendra*) became the predominant plant species in the drained lands, but at locations with re-introduced tidal flows, such as our study site, they died off and the original mangrove vegetation (*Avicennia marina, Aegiceras corniculatum*, and *Excoecaria agallocha*, etc…) returned (Newton et al., 2014). There have been several reports of various pentacyclic triterpenoid acids in *Melaleuca* species (Lee, 1998; Lee and Chang, 1998, 1999; Abdel Bar et al., 2008), the ursolic and oleanolic acids have also been found in mangrove leaves (Ghosh et al., 1985). However, due to their high reactivity, the abundance of these compounds in sediments is rare. The suite of compounds in the polar fractions of sites A1–A3 and the “Acidic Site” presented here may have been preserved by organo-mineral interactions with iron oxyhydroxides (Kogel-Knabner et al., 2008). Furthermore, previous research suggests that triterpenoids play an important role in salt adaptation for plants, and therefore the abundance of triterpenoids in mangrove species increases with salinity (Oku et al., 2003). Among the pentacyclic triterpenoids, betulin is more easily degraded and is thought to be a marker of mangrove *Avicennia* (Koch et al., 2005), which is widespread in the East Trinity study site (Department of Agriculture, Fisheries and Forestry, Queensland Government), consistent with our finding of abundant betulinic acid across our sites. Under reducing/anoxic conditions in sediments, pentacyclic triterpenoids are transformed by microbially-mediated A-ring degradation and progressive aromatization reactions during (early) diagenesis (e.g., Trendel et al., 1989; Le Métayer et al., 2005; Melendez et al., 2013; Schnell et al., 2014; Figure 2).

In addition to the type and abundance of organic matter, the existence of plants would also influence microbial distributions. In the rhizosphere area of treated wetlands, microbial diversity and activity are typically enhanced (Münch et al., 2005; Faulwetter et al., 2009). *Phragmites australis*, also called Common Reed, is distributed throughout the study site (Johns, 2010) with roots down to a depth of 20–30 cm (Stottmeister et al., 2003). It is reported that these roots improve nitrification and denitrification 20–50 mm away from the roots (Münch et al., 2005), and have a higher efficiency of transporting oxygen into the rhizosphere than diffusion alone (Armstrong and Armstrong, 1990). A higher redox potential gradient was observed from ~500 mV near root surface to ~−250 mV in 1–20 mm from the roots (Faulwetter et al., 2009). The roots likely increased soil heterogeneity in the subsurface, which could enhance microbial diversity since aerobic or microaerophilic microorganisms could survive in niches throughout otherwise anaerobic zones (Lamers et al., 2012).

### Alpha diversity controlled by organic matter, pH, and Eh values

Much research has shown that pH (Fierer and Jackson, 2006; Hartman et al., 2008; Lauber et al., 2009), Eh (DeAngelis et al., 2010), and organic matter content and type (Zhou et al., 2002) have strong influences on microbial diversity. In this study, a large range of alpha diversity indices was observed in the East Trinity wetland (Supplementary Table 1). The highest diversity was observed at A3 0–4 cm, and is comparable to that of a coral ecosystem (Chen et al., 2010; Gaidos et al., 2010). The lowest diversity was observed at A1 16–18 cm and is comparable with an AMD contaminated lake (Laplante and Derome, 2011). Both Eh and pH values led to differentiation of alpha diversity across the sampling sites.

Higher diversities were observed with more natural pH and higher Eh (more oxidizing) values (Figure 3). Site A1 contained much lower diversity (Supplementary Table 1, Supplementary Figure 1) when compared to sites A2 and A3, as a result of local pH and organic carbon content (Figure 8). The 0–8 cm depths of site A1 showed the lowest pH values (3–5) across all the samples, and site A1 contained the lowest organic carbon content among all 3 sites (Figure 8, Supplementary Table 1) (Burton et al., 2011). These conditions likely prevented colonization and growth by less acid-tolerant microbial groups. Site A3 showed pH > 6 for all depths (Figure 3), and microbial diversity decreased with depth in response to Eh (Supplementary Table 1, Supplementary Figure 1). For site A2, diversity appears to be influenced by both pH and Eh. From 0 to 10 cm depths in site A2, the diversity decreased with Eh/depth, and the pH (from 4 to ~6) did not show an effect on decreasing diversity. But for depths 10–20 cm in site A2, pH increased to >6, and the degree of diversity also increased, even though the environment was more reduced (i.e., deeper). Compared to site A1, which mostly experiences exposure to air, and site A3, which is mostly tidal-inundated, site A2 cut through two different soil layers (Figure 1A) and experiences the most oscillatory redox fluctuations, and also contained the highest amount of dissolved organic matter (Burton et al., 2011). The Eh values we measured represent the most oxidized potential since sediments were collected during the low tide period. The fluctuating redox potential results in higher diversity than would otherwise be present under more static chemical conditions (DeAngelis et al., 2010). Previous study of the same site showed sulfate reduction has the highest rate at site A2, which is controlled by dissolved organic matter content (Burton et al., 2011). The more neutral pH, high organic matter content, and oscillatory redox fluctuations therefore likely resulted in the increased microbial diversity in site A2.

### Beta diversity shaped by soil layering, water saturation, and pH

The soil in the organic horizon contains high concentrations of organic carbon, while the sulfuric horizon contains the actual acid sulfate soil, and the sulfidic horizon consists of potential acid sulfate soils (Hicks et al., 1999). Both Jaccard and the Yue and Clayton indices showed that microbial community similarity changed spatially across the East Trinity field site (Figure 4). Based on principal coordinate scaling and hierarchical clustering, three zones were distinguishable. Zone 1 included all of sites A1 and A2 from 2 to 10 cm in depth. Zone 2 consisted of site A3, 0–4 cm, and site A2, 0–2 cm, in depth. Site A2, depths 10–20 cm, and site A3, depths 4–20 cm, made up Zone 3. When we compare microbial diversity with *in situ* soil layering, water saturation, and pH values, the boundaries of these three zones were consistent with major variations in these environmental parameters (Figure 1). Zone 1 was located in the organic horizon; the upper part of Zone 1 (A1, 0–8 cm, and A2, 2–4 cm) had the lowest pH values at 3.0–5.5, and the remaining lower part of Zone 1 had pH values between 5.5 and 6.5 (Figure 4A). Zone 2 experienced the most disturbance from tidal activity, with pH values between 5.5 and 6.5. Below the organic horizon is the sulfuric horizon, which hosts Zone 3 in which all samples had pH values > 6 (Figure 4A), reflecting more than a decade of tidal inundation treatment (QASSIT, 2000). In this zone, the pH values have shown a increase from 3–4 to 3–8 and pyrite has accumulated up to 30 μmol/g (Johnston et al., 2011a).

### Geochemical parameters influence specific functional guilds

*Proteobacteria* comprised the most abundant phylum at East Trinity (Figure 5). The most abundant classes (in decreasing order) were *delta*-, *gamma*-, *alpha*-, and *beta*-*proteobacteria*. This ordering differs from a more typical soil community structure, which exhibits the ordering *alpha-, delta-, beta*-, and then *gamma*-*proteobacteria* (Spain et al., 2009). The *delta*- and *gamma*- classes showed much higher abundances in East Trinity, suggesting marine and acidity influences. The *deltaproteobacteria* were more abundant in marine-influenced sediments [Figure 6, Zone 2, corresponding to the upper parts of sites A2 (0–10 cm) and A3 (0–14 cm)], which is consistent with seawater as a source of sulfate for bacterial sulfate reduction and the predominance of sulfate-reducing bacteria (SRB) within the *delta* class (Rabus et al., 2013). In these samples, >45% of *deltaproteobacteria* were most closely related to members of order *Desulfobacterales*. The observed distribution of SRB at higher abundances in the upper depths at these sites also suggests a certain degree of oxygen tolerance to periods between tidal inundation (Canfield and Des Marais, 1991; Baumgartner et al., 2006), and/or possibly rapid changes in SRB activity with tidal fluctuation. The relative higher abundances of *gamma*- and *alpha-proteobacteria* in Zone 1 (Figure 6) are consistent with the microbial community composition observed in some AMD systems (Edwards et al., 2006; Brantner et al., 2014; Kamika and Momba, 2014), which are comparable to some CASS systems in terms of extreme acidification. Previous studies revealed that gamma-proteobacteria are more abundant at lower pH (Kuang et al., 2013; Fabisch et al., 2013). *Acidobacteria* (Lauber et al., 2009), which favors a low pH environment, was also present in relatively high abundance in Zone 1 but only in the higher pH area (Figure 6).

When the abundance of organic carbon exceeds the rate at which microorganisms can consume this resource (e.g., the maximum rate of enzymatic activity), microbes may not need to compete for electrons and carbon (Ling et al., 2012) and diversity can increase (Zhou et al., 2002). High organic matter can also increase soil aggregation by decreasing wetability (Chenu et al., 2000), which in turn further promotes physical heterogeneity and microbial diversity. The high concentration and multiple types of organic matter present at East Trinity could facilitate co-habitation of different metabolic guilds in close proximity within redox gradients throughout our sampling sites. For example, in Zone 1 (Figure 5), 49% of *beta-proteobacteria* were derived from the genus *Delftia* (Figure 5), which is known to possess nitrate reduction ability (Wen et al., 1999; Shigematsu et al., 2003) and was isolated from biofilm of common reed *P. australis* (Borsodi et al., 2007), a plant species common to the study area (Johns, 2010). Roughly 64% of *epsilon-proteobacteria* were closely related to *Sulfurimonas*, which possesses sulfur and thiosulfate oxidation abilities (Inagaki et al., 2003; Takai, 2006). Methanogenic *Methanomicrobia* showed increased abundance toward site A3 and *deltaproteobacteria*, typically associated with IRB and/or SRB, was a dominant class in all three sites. These metabolic guilds usually compete with each other for a limited energy source by maintaining the concentration of that source at the lowest threshold therefore establish a well resolved redox zonation which can be predicted thermodynamically (Hoehler et al., 1998).

The number of sequences representative of *deltaproteobacteria* was proportional (linear regression, *r*^2^ = 0.59, *p* = 0.002) to those representing known iron-reducing bacteria or close relatives for Zone 3 (at the genus level, Table 1), which represents the sulfuric horizon (Figure 7). Zone 1 did not show this proportionality (*r*^2^ = 0.17, *p* = 0.1427), and there are not enough samples in Zone 2 to demonstrate correlation convincingly. Site A3, depths 4–6 cm, differed from all other samples and can be explained by the observation that the sulfuric horizon is much closer to the tidal zone in site A3 than in site A2 (Figure 7). We infer that high organic content allowed the two metabolic guilds effectively to co-exist in Zone 3 (Figure 8). The (re)precipitation of iron sulfide minerals will thermodynamically favor iron, sulfate and elemental sulfur reduction, by removing the products of these metabolic reactions. In addition, tidal activity can potentially drive reductive dissolution of crystalline iron(III) minerals (e.g., jarosite) from the lower soil profile, redistributing the iron as poorly crystalline iron(III) minerals in the upper organic horizon (Johnston et al., 2011a). These poorly crystalline iron(III) minerals can act as a relatively labile electron acceptor for iron-reducing bacteria. This observation suggests that microorganisms exhibit a relatively rapid response to tidally generated redox fluctuations.

To test the central hypothesis of this study, a thermodynamic-kinetic model was used to evaluate factors that control microbial metabolic rates. Microorganisms conserve energy from redox changes between the reactant and the product to form adenosine triphosphate (ATP). When the energy available in the environment is in excess of energy conserved by microbial metabolism, the thermodynamic factor moves toward a greater value, which means the reaction is far from equilibrium and the reaction has a greater tendency to move in the forward direction. Taking acetotrophic sulfate reduction as an example, the concentrations of sulfate and organic carbon (reactants) are higher than the concentrations of sulfide and bicarbonate (products) in the study site, and higher thermodynamic factors (0.75–0.89) were observed (Figure 8). In the study site, sulfide was removed by deposition of iron-sulfide minerals and bicarbonate is removed by titrating acidity (Johnston et al., 2011b); both mechanisms favor sulfate reduction thermodynamically. In this situation, thermodynamic limitation can be ignored. The microbial metabolic rate law then becomes:

(13) $$v=k\left[X\right]\frac{\left[{\text{SO}}_{4}^{2-}\right]}{K_{{\text{SO}}_{4}^{2-}}+\left[{\text{SO}}_{4}^{2-}\right]}\frac{\left[{\text{CH}}_{3}{\text{COO}}^{-}\right]}{K_{{\text{CH}}_{3}{\text{COO}}^{-}}+\left[{\text{CH}}_{3}{\text{COO}}^{-}\right]}$$

In this case, the concentrations of sulfate and acetate were greater than the half-reaction constant *K*_SO^2−^_4__ and *K*_CH_3_COO^−^_ by at least one order of magnitude, and therefore the kinetic factor moves toward unity (Figure 8, Supplementary Table 2). This phenomenon was observed by Jin and Bethke (2003) for the initial stage of an incubation experiment when all substrates were present at high level. Both thermodynamic and kinetic factors showed high values in the study site, suggesting that the energy available was higher than the equilibrium state. The “snapshot” data used in this study support the hypothesis that organic matter content was higher than thermodynamic maintainance concentrations. Therefore, we infer that IRB and SRB did not need to compete for energy in the study site.

Microbial metabolic rates depend on a the rate constant *k* and microbial biomass concentrations. Biomass in the thermodynamic-kinetic model and other kinetic models does not account for dormant microbial cells (Jin et al., 2013). However, dormancy has been reported to help maintain biodiversity (Jones and Lennon, 2010), and was suggested as a survival strategy in highly dynamic environments (Lennon and Jones, 2011).

### Microbial ecology and CASS system evaluation

Microbial distributions in the East Trinity wetlands support the paradigm of community selection by from a homogenous population (De Wit and Bouvier, 2006), which is controlled by environmental heterogeneities (Bowen et al., 2009) associated with increased acid or salinity in this site. High concentrations and multiple types of organic matter would further increase similarity across samples. The low axis loading in our principal coordinate analysis (Figure 4A) confirmed that all sites were similar in terms of microbial community structure. Zones of similar dominant microbial guilds were defined across sites on the basis of environmental parameters such as pH, Eh, soil layering, and water saturation. This research revealed that vertically stratified models linking redox zonation and microbial guild distribution are not useful for predicting biogeochemical cycling at East Trinity.

Tidal re-inundation is being tested as an effective means for natural remediation of CASS systems. However, tidal fluctuations can make CASS systems highly dynamic environments with respect to redox states and the flux of nutrients and electron donors or acceptors. Correspondingly, microbial communities living in tidal zones experience both static and fluctuating environmental conditions that, in turn, are modulated by lithological compositions and hydrological connectivity. For researchers attempting to construct complete biogeochemical process models, these factors must also be considered.

### Conflict of interest statement

The authors declare that the research was conducted in the absence of any commercial or financial relationships that could be construed as a potential conflict of interest.

## References

[B1] Abdel Bar F. M. Zaghloul A. M. Bachawal S. V. Sylvester P. W. Ahmad K. F. Sayed El K. A. (2008). Antiproliferative triterpenes from *Melaleuca ericifolia*. J. Nat. Prod. 71, 1787–1790. 10.1021/np800360a 18826277

[B2] Alongi D. M. (2008). Mangrove forests: resilience, protection from tsunamis, and responses to global climate change. Estuar. Coast. Shelf Sci. 76, 1–13. 10.1016/j.ecss.2007.08.024

[B3] Armstrong J. Armstrong W. (1990). Light−enhanced convective throughflow increases oxygenation in rhizomes and rhizosphere of *Phragmites australis* (Cav.) Trin. ex Steud. New Phytol. 141, 121–128. 10.1111/j.1469-8137.1990.tb00382.x 33874300

[B4] Åström M. Björklund A. (1995). Impact of acid sulfate soils on stream water geochemistry in western Finland. J. Geochem. Explor. 55, 1–8. 10.1016/0375-6742(95)00018-6

[B5] Baumgartner L. K. Reid R. P. Dupraz C. Decho A. W. Buckley D. H. Spear J. R. (2006). Sulfate reducing bacteria in microbial mats: changing paradigms, new discoveries. Sediment. Geol. 185, 131–145. 10.1016/j.sedgeo.2005.12.008

[B6] Bethke C. (2007). Geochemical and Biogeochemical Reaction Modeling. New York, NY: Cambridge University Press 10.1017/cbo9780511619670

[B7] Borsodi A. K. Rusznyák A. Molnár P. Vladár P. Reskóné M. N. Tóth E. M.. (2007). Metabolic activity and phylogenetic diversity of reed (*Phragmites australis*) periphyton bacterial communities in a hungarian shallow soda lake. Microb. Ecol. 53, 612–620. 10.1007/s00248-006-9133-x 17406774

[B8] Bowen J. L. Crump B. C. Deegan L. A. Hobbie J. E. (2009). Salt marsh sediment bacteria: their distribution and response to external nutrient inputs. ISME J. 3, 924–934. 10.1038/ismej.2009.44 19421233

[B9] Brantner J. S. Haake Z. J. Burwick J. E. Menge C. M. Hotchkiss S. T. Senko J. M. (2014). Depth-dependent geochemical and microbiological gradients in Fe(III) deposits resulting from coal mine-derived acid mine drainage. Front. Microbiol. 5:215. 10.3389/fmicb.2014.00215 24860562 PMC4030175

[B10] Bronswijk J. J. B. Groenenberg J. E. Ritsema C. J. van Wijk A. L. M. Nugroho K. (1995). Evaluation of water management strategies for acid sulphate soils using a simulation model: a case study in Indonesia. Agric. Water Manage. 27, 125–142. 10.1016/0378-3774(95)01135-6

[B11] Brosius J. Dull T. J. Sleeter D. D. Noller H. F. (1981). Gene organization and primary structure of a ribosomal RNA operon from *Escherichia coli*. J. Mol. Biol. 148, 107–127. 10.1016/0022-2836(81)90508-8 7028991

[B12] Burton E. D. Bush R. T. Johnston S. G. Sullivan L. A. (2011). Sulfur biogeochemical cycling and novel Fe–S mineralization pathways in a tidally re-flooded wetland. Geochim. Cosmochim. Acta 75, 3434–3451. 10.1016/j.gca.2011.03.020

[B13] Burton E. D. Bush R. T. Sullivan L. A. Johnston S. G. Hocking R. K. (2008). Mobility of arsenic and selected metals during re-flooding of iron- and organic-rich acid-sulfate soil. Chem. Geol. 253, 64–73. 10.1016/j.chemgeo.2008.04.006

[B14] Caccavo F. Lonergan D. J. Lovley D. R. Davis M. Stolz J. F. McInerney M. J. (1994). *Geobacter sulfurreducens* sp. nov., a hydrogen- and acetate-oxidizing dissimilatory metal-reducing microorganism. Appl. Environ. Microbiol. 60, 3752–3759. 7527204 10.1128/aem.60.10.3752-3759.1994 PMC201883

[B15] Canfield D. Des Marais D. (1991). Aerobic sulfate reduction in microbial mats. Science 251, 1471–1473. 10.1126/science.11538266 11538266

[B16] Chapelle F. H. Lovley D. R. (1992). Competitive exclusion of sulfate reduction by Fe(lll)-reducing bacteria: a mechanism for producing discrete zones of high-iron ground water. Ground Water 30, 29–36. 10.1111/j.1745-6584.1992.tb00808.x

[B17] Chen C.-P. Tseng C.-H. Chen C. A. Tang S.-L. (2010). The dynamics of microbial partnerships in the coral Isopora palifera. ISME J. 5, 728–740. 10.1038/ismej.2010.151 20962876 PMC3105734

[B18] Chenu C. Le Bissonnais Y. Arrouays D. (2000). Organic matter influence on clay wettability and soil aggregate stability. Soil Sci. Soc. Am. J. 64, 1479–1486. 10.2136/sssaj2000.6441479x

[B19] Claesson M. J. O'Sullivan O. Wang Q. Nikkilä J. Marchesi J. R. Smidt H.. (2009). Comparative analysis of pyrosequencing and a phylogenetic microarray for exploring microbial community structures in the human distal intestine. PLoS ONE 4:e6669. 10.1371/journal.pone.0006669 19693277 PMC2725325

[B20] Coates J. D. Ellis D. J. Gaw C. V. Lovley D. R. (1999). Geothrix fermentans gen. nov., sp. nov., a novel Fe(III)-reducing bacterium from a hydrocarbon-contaminated aquifer. Int. J. Syst. Bacteriol. 49(Pt 4), 1615–1622. 10.1099/00207713-49-4-1615 10555343

[B21] Coates J. D. Lonergan D. J. Philips E. J. Jenter H. Lovley D. R. (1995). *Desulfuromonas palmitatis* sp. nov., a marine dissimilatory Fe(III) reducer that can oxidize long-chain fatty acids. Arch. Microbiol. 164, 406–413. 10.1007/BF02529738 8588742

[B22] Crammond N. (2002). The occurrence of thaumasite in modern construction – a review. Cement Concrete Comp. 24, 393–402. 10.1016/S0958-9465(01)00092-0

[B23] Cummings D. E. Caccavo F. Jr. Spring S. Rosenzweig R. F. (1999). *Ferribacterium limneticum*, gen. nov., sp. nov., an Fe(III)-reducing microorganism isolated from mining-impacted freshwater lake sediments. Arch. Microbiol. 171, 183–188. 10.1007/s002030050697

[B24] De Wit R. Bouvier T. (2006). “Everything is everywhere, but, the environment selects;” what did Baas Becking and Beijerinck really say? Environ. Microbiol. 8, 755–758. 10.1111/j.1462-2920.2006.01017.x 16584487

[B25] DeAngelis K. M. Silver W. L. Thompson A. W. Firestone M. K. (2010). Microbial communities acclimate to recurring changes in soil redox potential status. Environ. Microbiol. 12, 3137–3149. 10.1111/j.1462-2920.2010.02286.x 20629704

[B26] Denmead O. T. Macdonald B. C. T. Bryant G. White I. Wang W. Moody P. (2007). Greenhouse gas emissions from sugarcane soils and nitrogen fertiliser management: II, in Proceedings of the 2006 Conference of the Australian Society of Sugar Cane Technologists held at Mackay, Queensland, Australia, 2-5 May 2006, 252-260 27, 97–105.

[B27] Dent D. (1986). Acid Sulphate Soils: a Baseline for Research and Development. Wageningen: ILRI Publications.

[B28] Dent D. Pons L. (1995). A world perspective on acid sulphate soils. Geoderma 67, 263–276. 10.1016/0016-7061(95)00013-E

[B29] Edgar R. C. Haas B. J. Clemente J. C. Quince C. Knight R. (2011). UCHIME improves sensitivity and speed of chimera detection. Bioinformatics 27, 2194–2200. 10.1093/bioinformatics/btr381 21700674 PMC3150044

[B30] Edwards R. A. Rodriguez-Brito B. Wegley L. Haynes M. Breitbart M. Peterson D. M.. (2006). Using pyrosequencing to shed light on deep mine microbial ecology. BMC Genomics 7:57. 10.1186/1471-2164-7-57 16549033 PMC1483832

[B31] Eiserbeck C. Nelson R. K. Grice K. Curiale J. (2012). Comparison of GC–MS, GC–MRM-MS, and GC× GC to characterise higher plant biomarkers in Tertiary oils and rock extracts. Geochim. Cosmochim. Acta 87, 299–322. 10.1016/j.gca.2012.03.033

[B32] Fabisch M. Beulig F. Akob D. M. Küsel K. (2013). Surprising abundance of Gallionella-related iron oxidizers in creek sediments at pH 4.4 or at high heavy metal concentrations. Front. Microbiol. 4:390. 10.3389/fmicb.2013.00390 24385973 PMC3866512

[B33] Faulwetter J. L. Gagnon V. Sundberg C. Chazarenc F. Burr M. D. Brisson J. (2009). Microbial processes influencing performance of treatment wetlands: a review. Ecol. Eng. 35, 987–1004. 10.1016/j.ecoleng.2008.12.030

[B34] Fierer N. Jackson R. B. (2006). The diversity and biogeography of soil bacterial communities. Proc. Natl. Acad. Sci. U.S.A. 103, 626–631. 10.1073/pnas.0507535103 16407148 PMC1334650

[B35] Finneran K. T. Forbush H. M. VanPraagh C. V. G. Lovley D. R. (2002). *Desulfitobacterium metallireducens* sp. nov., an anaerobic bacterium that couples growth to the reduction of metals and humic acids as well as chlorinated compounds. Int. J. Syst. Evol. Microbiol. 52, 1929–1935. 10.1099/ijs.0.02121-0 12508850

[B36] Fitzpatrick R. W. (2003). Overview of acid sulfate soil properties, environmental hazards, risk mapping and policy development in Australia, in Advances in Regolith Proceedings of the CRC LEME Regional Regolith Symposia (Bentley, WA: CRC LEME).

[B37] Gaidos E. Rusch A. Ilardo M. (2010). Ribosomal tag pyrosequencing of DNA and RNA from benthic coral reef microbiota: community spatial structure, rare members and nitrogen-cycling guilds. Environ. Microbiol. 13, 1138–1152. 10.1111/j.1462-2920.2010.02392.x 21176054

[B38] Ghosh A. Misra S. Dutta A. K. Choudhury A. (1985). Pentacyclic triterpenoids and sterols from seven species of mangrove. Phytochemistry 24, 1725–1727. 10.1016/S0031-9422(00)82541-8

[B39] Golyshina O. V. Pivovarova T. A. Karavaiko G. I. Kondratéva T. F. Moore E. R. Abraham W. R.. (2000). *Ferroplasma acidiphilum* gen. nov., sp. nov., an acidophilic, autotrophic, ferrous-iron-oxidizing, cell-wall-lacking, mesophilic member of the Ferroplasmaceae fam. nov., comprising a distinct lineage of the Archaea. Int. J. Syst. Evol. Microbiol. 50(Pt 3), 997–1006. 10.1099/00207713-50-3-997 10843038

[B40] Hartman W. H. Richardson C. J. Vilgalys R. Bruland G. L. (2008). Environmental and anthropogenic controls over bacterial communities in wetland soils. Proc. Natl. Acad. Sci. U.S.A. 105, 17842–17847. 10.1073/pnas.0808254105 19004771 PMC2584698

[B41] Hicks W. Bowman G. Fitzaptrick R. W. (1999). East Trinity Acid Sulfate Soils: Part 1: Environmental Hazards. Adelaide: CSIRO Publishing.

[B42] Hoehler T. M. Alperin M. J. Albert D. B. Martens C. S. (1998). Thermodynamic control on hydrogen concentrations in anoxic sediments. Geochim. Cosmochim. Acta 62, 1745–1756. 10.1016/S0016-7037(98)00106-9

[B43] Inagaki F. Takai K. Kobayashi H. Nealson K. H. Horikoshi K. (2003). *Sulfurimonas autotrophica* gen. nov., sp. nov., a novel sulfur-oxidizing epsilon-proteobacterium isolated from hydrothermal sediments in the Mid-Okinawa Trough. Int. J. Syst. Evol. Microbiol. 53, 1801–1805. 10.1099/ijs.0.02682-0 14657107

[B44] Itoh T. Yamanoi K. Kudo T. Ohkuma M. Takashina T. (2011). *Aciditerrimonas ferrireducens* gen. nov., sp. nov., an iron-reducing thermoacidophilic actinobacterium isolated from a solfataric field. Int. J. Syst. Evol. Microbiol. 61, 1281–1285. 10.1099/ijs.0.023044-0 20639230

[B45] Jin Q. Bethke C. M. (2003). A new rate law describing microbial respiration. Appl. Environ. Microbiol. 69, 2340–2348. 10.1128/AEM.69.4.2340-2348.2003 12676718 PMC154818

[B46] Jin Q. Bethke C. M. (2005). Predicting the rate of microbial respiration in geochemical environments. Geochim. Cosmochim. Acta 69, 1133–1143. 10.1016/j.gca.2004.08.010

[B47] Jin Q. Bethke C. M. (2007). The thermodynamics and kinetics of microbial metabolism. Am. J. Sci. 307, 643–677. 10.2475/04.2007.01

[B48] Jin Q. Bethke C. M. (2009). Cellular energy conservation and the rate of microbial sulfate reduction. Geology 37, 1027–1030. 10.1130/G30185A.1

[B49] Jin Q. Roden E. E. Giska J. R. (2013). Geomicrobial kinetics: extrapolating laboratory studies to natural environments. Geomicrobiol. J. 30, 173–185. 10.1080/01490451.2011.653084

[B50] Johns L. (2010). Field Guide to Common Saltmarsh Plants of Queensland. City East: Department of Employment, Economic Development and Innovation.

[B51] Johnston S. Keene A. Bush R. (2009b). Remediation of coastal acid sulfate soils by tidal inundation: effectiveness and geochemical implications, in Proceedings of 18th NSW Coastal Conference, Ballina, NSW, 3-6 November, East Coast Conferences (Coffs Harbour, NSW).

[B52] Johnston S. G. Bush R. T. Sullivan L. A. Burton E. D. Smith D. Martens M. A. (2009a). Changes in water quality following tidal inundation of coastal lowland acid sulfate soil landscapes. Estuar. Coastal Self Sci. 81, 257–266. 10.1016/j.ecss.2008.11.002

[B53] Johnston S. G. Keene A. F. Burton E. D. Bush R. T. Sullivan L. A. (2012). Quantifying alkalinity generating processes in a tidally remediating acidic wetland. Chem. Geol. 304–305, 106–116. 10.1016/j.chemgeo.2012.02.008

[B54] Johnston S. G. Keene A. F. Burton E. D. Bush R. T. Sullivan L. A. McElnea A.. (2010). Arsenic mobilization in a seawater inundated acid sulfate soil. Environ. Sci. Technol. 44, 1968–1973. 10.1021/es903114z 20155899

[B55] Johnston S. G. Keene A. F. Bush R. T. Burton E. D. Sullivan L. A. Isaacson L. (2011a). Iron geochemical zonation in a tidally inundated acid sulfate soil wetland. Chem. Geol. 280, 257–270. 10.1016/j.chemgeo.2010.11.014

[B56] Johnston S. G. Keene A. F. Bush R. T. Sullivan L. A. Wong V. N. L. (2011b). Tidally driven water column hydro-geochemistry in a remediating acidic wetland. J. Hydrol. 409, 128–139. 10.1016/j.jhydrol.2011.08.010

[B57] Jones S. E. Lennon J. T. (2010). Dormancy contributes to the maintenance of microbial diversity. Proc. Natl. Acad. Sci. U.S.A. 107, 5881–5886. 10.1073/pnas.0912765107 20231463 PMC2851880

[B58] Kamika I. Momba M. N. B. (2014). Microbial diversity of Emalahleni mine water in South Africa and tolerance ability of the predominant organism to vanadium and nickel. PLoS ONE 9:e86189. 10.1371/journal.pone.0086189 24465951 PMC3899216

[B59] Khan S. T. Harayama S. (2007). *Paraferrimonas sedimenticola* gen. nov., sp. nov., a marine bacterium of the family Ferrimonadaceae. Int. J. Syst. Evol. Microbiol. 57, 1493–1498. 10.1099/ijs.0.64529-0 17625182

[B60] Koch B. P. Harder J. Lara R. J. Kattner G. (2005). The effect of selective microbial degradation on the composition of mangrove derived pentacyclic triterpenols in surface sediments. Org. Geochem. 36, 273–285. 10.1016/j.orggeochem.2004.07.019

[B61] Kogel-Knabner I. Guggenberge G. Kleber M. Kandeler E. Kalbitz K. Scheu S. (2008). Organo-mineral associations in temperate soils: integrating biology, mineralogy, and organic matter chemistry. J. Plant Nutr. Soil Sci. 171, 61–82. 10.1002/jpln.200700048

[B62] Kristensen E. Bouillon S. Dittmar T. Marchand C. (2008). Organic carbon dynamics in mangrove ecosystems: a review. Aquat. Bot. 89, 201–219. 10.1016/j.aquabot.2007.12.005

[B63] Kuang J.-L. Huang L.-N. Chen L.-X. Hua Z.-S. Li S.-J. Hu M.. (2013). Contemporary environmental variation determines microbial diversity patterns in acid mine drainage. ISME J. 7, 1038–1050. 10.1038/ismej.2012.139 23178673 PMC3635239

[B64] Lamers L. P. van Diggelen J. M. Op den Camp H. J. Visser E. J. Lucassen E. C. Vile M. A. (2012). Microbial transformations of nitrogen, sulfur, and iron dictate vegetation composition in wetlands: a review. Front. Microbiol. 3:156 10.3389/fmicb.2012.00156 PMC3336091 22539932

[B65] Laplante K. Derome N. (2011). Parallel changes in the taxonomical structure of bacterial communities exposed to a similar environmental disturbance. Ecol. Evol. 1, 489–501. 10.1002/ece3.37 22393517 PMC3287327

[B66] Lauber C. L. Hamady M. Knight R. Fierer N. (2009). Pyrosequencing-based assessment of soil pH as a predictor of soil bacterial community structure at the continental scale. Appl. Environ. Microbiol. 75, 5111–5120. 10.1128/AEM.00335-09 19502440 PMC2725504

[B67] Le Métayer P. Schaeffer P. Duringer P. Roussé S. (2005). 4, 4'-Dimethyldinaphtho [a, d] cycloheptane, a naturally occurring polyaromatic derivative related to triterpenoids of the serratane series. Org. Lett. 7, 3041–3044. 10.1021/ol0509944 15987200

[B68] Lee C. Chang M. (1998). A new norlupene from the leaves of *Melaleuca leucadendron*. J. Nat. Prod. 61, 375–376. 10.1021/np9606052 9548878

[B69] Lee C. Chang M. (1999). Four new triterpenes from the heartwood of *Melaleuca leucadendron*. J. Nat. Prod. 62, 1003–1005. 10.1021/np980169e 10425126

[B70] Lee C. K. (1998). Ursane triterpenoids from leaves of *Melaleuca leucadendron*. Phytochemistry 49, 1119–1122. 10.1016/S0031-9422(97)01061-3

[B71] Lennon J. T. Jones S. E. (2011). Microbial seed banks: the ecological and evolutionary implications of dormancy. Nat. Rev. Microbiol. 9, 119–130. 10.1038/nrmicro2504 21233850

[B72] Ling Y. C. Chen Y. J. Sun C. H. Cheng T. W. Wang P. L. (2012). Potential of microbial methane formation in a high-temperature hydrocarbon seep. Appl. Geochem. 27, 1666–1678. 10.1016/j.apgeochem.2012.04.002

[B73] Ljung K. Maley F. Cook A. Weinstein P. (2009). Acid sulfate soils and human health—A millennium ecosystem assessment. Environ. Sci. Technol. 35, 1234–1242. 10.1016/j.envint.2009.07.002 19647876

[B74] Lovley D. R. Klug M. J. (1982). Intermediary metabolism of organic matter in the sediments of a eutrophic lake. Appl. Environ. Microbiol. 43, 552–560. 16345963 10.1128/aem.43.3.552-560.1982 PMC241873

[B75] Lovley D. R. Phillips E. J. (1987). Competitive mechanisms for inhibition of sulfate reduction and methane production in the zone of ferric iron reduction in sediments. Appl. Environ. Microbiol. 53, 2636–2641. 16347483 10.1128/aem.53.11.2636-2641.1987 PMC204165

[B76] Lovley D. R. Giovannoni S. J. White D. C. Champine J. E. Phillips E. J. Gorby Y. A.. (1993). *Geobacter metallireducens* gen. nov. sp. nov., a microorganism capable of coupling the complete oxidation of organic compounds to the reduction of iron and other metals. Arch. Microbiol. 159, 336–344. 10.1007/BF00290916 8387263

[B77] Lovley D. R. Phillips E. J. Lonergan D. J. Widman P. K. (1995). Fe(III) and S0 reduction by Pelobacter carbinolicus. Appl. Environ. Microbiol. 61, 2132–2138. 7793935 10.1128/aem.61.6.2132-2138.1995 PMC167486

[B78] Macdonald B. Denmead O. T. White I. (2004). Natural sulfur dioxide emissions from sulfuric soils. Atmos. Environ. 38, 1473–1480. 10.1016/j.atmosenv.2003.12.005

[B79] Melendez I. Grice K. Schwark L. (2013). Exceptional preservation of Palaeozoic steroids in a diagenetic continuum. Sci. Rep. 3:2768. 10.1038/srep02768 24067597 PMC3783881

[B80] Michaelis L. Menten M. L. Johnson K. A. Goody R. S. (2011). The original michaelis constant: translation of the 1913 Michaelis-Menten paper. Biochemistry 50, 8264–8269. 10.1021/bi201284u 21888353 PMC3381512

[B81] Monod J. (1949). The growth of microbial cultures. Annu. Rev. Microbiol. 3, 371–394. 10.1146/annurev.mi.03.100149.0021034169194

[B82] Moreau J. W. Fournelle J. H. Banfield J. F. (2013). Quantifying heavy metals sequestration by sulfate-reducing bacteria in an Acid mine drainage-contaminated natural wetland. Front. Microbiol. 4:43. 10.3389/fmicb.2013.00043 23487496 PMC3594707

[B83] Münch C. Kuschk P. Röske I. (2005). Root stimulated nitrogen removal: only a local effect or important for water treatment? Water Sci. Technol. 51, 185–192. 16042258

[B84] Nabbefeld B. Grice K. Twitchett R. J. Summons R. E. Hays L. Bottcher M. E. (2010). An integrated biomarker, isotopic and palaeoenvironmental study through the Late Permian event at Lusitaniadalen, Spitsbergen. Earth Planet Sci. Lett. 291, 84–96. 10.1016/j.epsl.2009.12.053

[B85] Newton R. M. Addicott P. E. Bannink P. (2014). Vegetation Survey of the East Trinity Reclamation Site. Brisbane, QLD: Queensland Herbarium, Queensland Department of Science, Information Technology, Innovation and the Arts.

[B86] Ohba H. Owa N. (2005). Vertical distribution of physico-chemical properties and number of sulfur-oxidizing bacteria in the buried layer of soil profiles with marine-reduced sulfur compounds. Soil Sci. Plant Nutr. 51, 379–388. 10.1111/j.1747-0765.2005.tb00043.x

[B87] Oku H. Baba S. Koga H. Takara K. Iwasaki H. (2003). Lipid composition of mangrove and its relevance to salt tolerance. J. Plant Res. 116, 37–45. 10.1007/s10265-002-0069-z 12605298

[B89] Pollock J. Weber K. A. Lack J. Achenbach L. A. Mormile M. R. Coates J. D. (2007). Alkaline iron(III) reduction by a novel alkaliphilic, halotolerant, Bacillus sp. isolated from salt flat sediments of Soap Lake. Appl. Microbiol. Biotechnol. 77, 927–934. 10.1007/s00253-007-1220-5 17943280

[B90] Powell B. Martens M. (2005). A review of acid sulfate soil impacts, actions and policies that impact on water quality in Great Barrier Reef catchments, including a case study on remediation at East Trinity. Mar. Pollut. Bull. 51, 149–164. 10.1016/j.marpolbul.2004.10.047 15757717

[B91] QASSIT Q. A. S. S. I. T. (2000). East Trinity Property Acid Sulfate Soils Remediation Action Plan. Indooroopilly: Queensland Government.

[B92] Rabus R. Hansen T. A. Widdel F. (2013). Dissimilatory sulfate- and sulfur-reducing prokaryotes, in The Prokaryotes, eds Rosenberg E. DeLong E. F. Lory S. Stackebrandt E. Thompson F. (Berlin; Heidelberg: Springer), 309–404.

[B93] Robertson W. J. Bowman J. P. Franzmann P. D. Mee B. J. (2001). *Desulfosporosinus meridiei* sp. nov., a spore-forming sulfate-reducing bacterium isolated from gasolene-contaminated groundwater. Int. J. Syst. Evol. Microbiol. 51, 133–140. 11211250 10.1099/00207713-51-1-133

[B94] Roh Y. Gao H. Vali H. Kennedy D. W. Yang Z. K. Gao W.. (2006). Metal reduction and iron biomineralization by a psychrotolerant Fe(III)-reducing bacterium, Shewanella sp. strain PV-4. Appl. Environ. Microbiol. 72, 3236–3244. 10.1128/AEM.72.5.3236-3244.2006 16672462 PMC1472395

[B95] Rosicky M. A. Sullivan L. A. Slavich P. G. Hughes M. (2004). Factors contributing to the acid sulfate soil scalding process in the coastal floodplains of New South Wales, Australia. Aust. J. Soil Res. 42, 587–594. 10.1071/SR03076

[B96] Rosselló-Mora R. A. Ludwig W. Kampfer P. (1995). *Ferrimonas balearica* gen. nov., spec. nov., a New Marine Facultative Fe(III)-reducing Bacterium. Syst. Appl. Microbiol. 18, 196–202. 10.1016/S0723-2020(11)80390-5

[B97] Sammut J. White I. Melville M. D. (1996). Acidification of an estuarine tributary in eastern Australia due to drainage of acid sulfate soils. Mar. Freshwater Res. 47, 669–684. 10.1071/MF9960669

[B98] Sawadogo J. B. Traoré A. S. Dianou D. (2013). Relationships between methanogens and sulfate-reducing bacteria during acetate, formate and lactate metabolism in *Macrotermes bellicosus* Termite Gut. World Appl. Sci. J. 24, 154–162. 10.5829/idosi.wasj.2013.24.02.2215

[B99] Schloss P. D. (2010). The effects of alignment quality, distance calculation method, sequence filtering, and region on the analysis of 16S rRNA gene-based studies. PLoS Comput. Biol. 6:e1000844. 10.1371/journal.pcbi.1000844 20628621 PMC2900292

[B100] Schloss P. D. Gevers D. Westcott S. L. (2011). Reducing the effects of PCR amplification and sequencing artifacts on 16S rRNA-based studies. PLoS ONE 6:e27310. 10.1371/journal.pone.0027310 22194782 PMC3237409

[B101] Schloss P. D. Westcott S. L. Ryabin T. Hall J. R. Hartmann M. Hollister E. B.. (2009). Introducing mothur: open-source, platform-independent, community-supported software for describing and comparing microbial communities. Appl. Environ. Microbiol. 75, 7537–7541. 10.1128/AEM.01541-09 19801464 PMC2786419

[B102] Schnell G. Schaeffer P. Tardivon H. Motsch E. (2014). Contrasting diagenetic pathways of higher plant triterpenoids in buried wood as a function of tree species. Org. Geochem. 66, 107–124. 10.1016/j.orggeochem.2013.11.001

[B103] Shigematsu T. Yumihara K. Ueda Y. Numaguchi M. Morimura S. Kida K. (2003). *Delftia tsuruhatensis* sp. nov., a terephthalate-assimilating bacterium isolated from activated sludge. Int. J. Syst. Evol. Microbiol. 53, 1479–1483. 10.1099/ijs.0.02285-0 13130036

[B104] Simek M. Virtanen S. Simojoki A. Chroòáková A. Elhottová D. Krištùfek V.. (2013). The microbial communities and potential greenhouse gas production in boreal acid sulphate, non-acid sulphate, and reedy sulphidic soils. Sci. Total Environ. 466C–467C, 663–672. 23962436 10.1016/j.scitotenv.2013.07.083

[B105] Spain A. M. Krumholz L. R. Elshahed M. S. (2009). Abundance, composition, diversity and novelty of soil Proteobacteria. ISME J. 3, 992–1000. 10.1038/ismej.2009.43 19404326

[B106] Statistical Package R. (2009). R: A Language and Environment for Statistical Computing. Vienna: R Foundation for Statistical Computing.

[B107] Stephens F. J. Ingram M. (2006). Two cases of fish mortality in low pH, aluminium rich water. J. Fish Dis. 29, 765–770. 10.1111/j.1365-2761.2006.00772.x 17169109

[B108] Stottmeister U. Wießner A. Kuschk P. Kappelmeyer U. Kästner M. Bederski O.. (2003). Effects of plants and microorganisms in constructed wetlands for wastewater treatment. Biotechnol. Adv. 22, 93–117. 10.1016/j.biotechadv.2003.08.010 14623046

[B109] Takai K. (2006). *Sulfurimonas paralvinellae* sp. nov., a novel mesophilic, hydrogen- and sulfur-oxidizing chemolithoautotroph within the Epsilonproteobacteria isolated from a deep-sea hydrothermal vent polychaete nest, reclassification of *Thiomicrospira denitrificans* as *Sulfurimonas denitrificans* comb. nov. and emended description of the genus Sulfurimonas. Int. J. Syst. Evol. Microbiol. 56, 1725–1733. 10.1099/ijs.0.64255-0 16901999

[B110] Temple K. L. Colmer A. R. (1951). The autotrophic oxidation of iron by a new bacterium, thiobacillus ferrooxidans. J. Bacteriol. 62, 605–611. 14897836 10.1128/jb.62.5.605-611.1951 PMC386175

[B111] Thauer R. K. Jungermann K. Decker K. (1977). Energy conservation in chemotrophic anaerobic bacteria. Bacteriol. Rev. 41, 100–180. 860983 10.1128/br.41.1.100-180.1977 PMC413997

[B112] Trendel J. M. Lohmann F. Kintzinger J. P. Albrecht P. Chiarone A. Riche C. (1989). Identification of des-A-triterpenoid hydrocarbons occurring in surface sediments. Tetrahedron 45, 4457–4470. 10.1016/S0040-4020(01)89081-5

[B113] Van Breemen N. (1973). Dissolved aluminum in acid sulfate soils and in acid mine waters. Soil Sci. Soc. Am. J. 37, 694–697. 10.2136/sssaj1973.03615995003700050020x20523977

[B114] Vandieken V. (2006). *Desulfuromonas svalbardensis* sp. nov. and *Desulfuromusa ferrireducens* sp. nov., psychrophilic, Fe(III)-reducing bacteria isolated from Arctic sediments, Svalbard. Int. J. Syst. Evol. Microbiol. 56, 1133–1139. 10.1099/ijs.0.63639-0 16627667

[B115] Wang Q. Garrity G. M. Tiedje J. M. Cole J. R. (2007). Assessment of the microbial ecology of ruminal methanogens in cattle with different feed efficiencies. Appl. Environ. Microbiol. 73, 5261–5267. 10.1128/AEM.00062-07 19717632 PMC2765141

[B116] Ward N. J. Shepherd T. Wang Z. (2014). Changes in the Surface Water Chemistry at Low Tide in Drainage Channels at East Trinity Coastal Wetland, Cairns, (August 2013). Lismore: Southern Cross University.

[B117] Warnes G. R. Bolker B. Bonebakker L. Gentleman R. Huber W. Liaw A.. (2009). gplots: various R programming tools for plotting data. R Package Version 2. 25208325

[B118] Wen A. Fegan M. Hayward C. Chakraborty S. Sly L. I. (1999). Phylogenetic relationships among members of the Comamonadaceae, and description of *Delftia acidovorans* (den Dooren de Jong 1926 and Tamaoka et al. 1987) gen. nov., comb. nov. Int. J. Syst. Bacteriol. 49, 567–576. 10.1099/00207713-49-2-567 10319477

[B119] White I. Melville M. Macdonald B. Quirk R. Hawken R. Tunks M. (2007). From conflicts to wise practice agreement and national strategy: cooperative learning and coastal stewardship in estuarine floodplain management, Tweed River, eastern Australia. J. Clean. Prod. 15, 1545–1558. 10.1016/j.jclepro.2006.07.049

[B120] White I. Melville M. D. Wilson B. P. Sammut J. (1997). Reducing acidic discharges from coastal wetlands in eastern Australia. Wetlands Ecol. Manag. 5, 55–72. 10.1023/A:1008227421258

[B121] Willett L. R. Crockford R. H. Milnes A. R. (1992). Transformations of iron, manganese and aluminium during oxidation of a sul?dic material from an acid sulfate soil. Catena Suppl. 21, 287–302.

[B122] Wu X. Wong Z. L. Sten P. Engblom S. Osterholm P. Dopson M. (2013). Microbial community potentially responsible for acid and metal release from an Ostrobothnian acid sulfate soil. FEMS Microbiol. Ecol. 84, 555–563. 10.1111/1574-6941.12084 23369102 PMC3732381

[B123] Young B. M. Harvey E. L. (1996). A spatial analysis of the relationship between mangrove (Avicennia marinavar. Australasica) physiognomy and sediment accretion in the Hauraki plains, New Zealand. Estuarine Coast. Shelf Sci. 42, 231–246. 10.1006/ecss.1996.0017

[B124] Yue J. C. Clayton M. K. (2005). A similarity measure based on species proportions. Commun. Stat. Theory Methods 34, 2123–2131. 10.1080/STA-20006641823236910

[B125] Zhou J. Xia B. Treves D. S. Wu L.-Y. Marsh T. L. O'Neill R. V.. (2002). Spatial and resource factors influencing high microbial diversity in soil. Appl. Environ. Microbiol. 68, 326–334. 10.1128/AEM.68.1.326-334.2002 11772642 PMC126564

