# Supplementary material for: Distribution of iron- and sulfate-reducing bacteria across a coastal acid sulfate soil (CASS) environment: implications for passive bioremediation by tidal inundation
Source: Front Microbiol. 2015 Jul 3;6:624. doi: 10.3389/fmicb.2015.00624 (PMC4490247; doi:10.3389/fmicb.2015.00624)
Supplement: Supplementary file 1 [file Table1.DOCX]

***Supplementary Material***

**Distribution of iron- and sulfate-reducing bacteria across a coastal acid sulfate soil (CASS) environment: implications for passive bioremediation by tidal inundation**

**Yu-Chen Ling^1,^*, Richard Bush^2^, Kliti Grice^3^, Svenja Tulipani^3^, Lyndon Berwick^3^ and John W. Moreau^1,^***

^1^School of Earth Sciences, University of Melbourne, Melbourne, VIC, Australia

^2^Southern Cross GeoScience, Southern Cross University, Lismore, NSW, Australia

^3^Western Australia Organic and Isotope Geochemistry Centre, Department of Chemistry, the Institute for Geoscience Research, Curtin University, Perth, WA, Australia

***Correspondence:** Yu-Chen Ling, School of Earth Sciences, University of Melbourne, Parkville, VIC 3010, Australia.

lchacol@gmail.com; John W. Moreau, School of Earth Sciences, University of Melbourne, Parkville, VIC 3010, Australia, jmoreau@unimelb.edu.au

1. **Supplementary Data**
2. **Supplementary Figures and Tables**

## Supplementary Tables

**Supplementary Table 1.** Information summary of sequences resampled to 1498 reads.

| **Name** | coverage | no. of observed OTUs | Chao | inverse Simpson | Simpson evenness | Shannon |
| --- | --- | --- | --- | --- | --- | --- |
| **A1, 0-2cm** | 80.97 | 454 | 1128.50 | 79.93 | 0.15 | 5.19 |
| **A1, 2-4cm** | 82.71 | 419 | 966.72 | 75.48 | 0.16 | 5.05 |
| **A1, 4-6cm** | 90.25 | 301 | 471.73 | 48.50 | 0.14 | 4.58 |
| **A1, 6-8cm** | 97.33 | 94 | 135.05 | 7.96 | 0.08 | 2.57 |
| **A1, 8-10cm** | 79.57 | 477 | 1325.45 | 81.82 | 0.14 | 5.26 |
| **A1, 10-12cm** | 84.91 | 394 | 832.36 | 55.09 | 0.12 | 4.94 |
| **A1, 12-14cm** | 91.26 | 245 | 430.11 | 18.91 | 0.07 | 3.87 |
| **A1, 14-16cm** | 85.05 | 378 | 808.62 | 63.80 | 0.14 | 4.94 |
| **A1, 16-18cm** | 97.66 | 73 | 127.09 | 6.37 | 0.08 | 2.28 |
| **A1, 18-20cm** | 93.12 | 213 | 338.07 | 19.50 | 0.08 | 3.82 |
|  |  |  |  |  |  |  |
| **A2, 0-2cm** | 67.22 | 737 | 1707.12 | 331.53 | 0.37 | 6.14 |
| **A2, 2-4cm** | 80.91 | 466 | 1093.00 | 106.03 | 0.20 | 5.30 |
| **A2, 4-6cm** | 90.19 | 277 | 515.47 | 21.29 | 0.07 | 4.24 |
| **A2, 6-8cm** | 86.58 | 332 | 718.54 | 34.22 | 0.09 | 4.44 |
| **A2, 8-10cm** | 88.52 | 332 | 585.55 | 74.68 | 0.20 | 4.86 |
| **A2, 10-12cm** | 79.64 | 467 | 1280.33 | 26.30 | 0.05 | 4.89 |
| **A2, 12-14cm** | 75.50 | 559 | 1442.70 | 76.19 | 0.11 | 5.43 |
| **A2, 14-16cm** | 81.24 | 476 | 1054.53 | 113.53 | 0.21 | 5.37 |
| **A2, 16-18cm** | 77.70 | 534 | 1295.79 | 142.00 | 0.23 | 5.56 |
| **A2, 18-20cm** | 76.84 | 543 | 1332.88 | 108.98 | 0.16 | 5.51 |
|  |  |  |  |  |  |  |
| **A3, 0-2cm** | 53.20 | 880 | 3668.07 | 278.50 | 0.25 | 6.27 |
| **A3, 2-4cm** | 56.48 | 880 | 2678.53 | 500.24 | 0.45 | 6.41 |
| **A3, 4-6cm** | 69.43 | 615 | 2010.37 | 64.59 | 0.09 | 5.35 |
| **A3, 6-8cm** | 69.49 | 685 | 1686.88 | 261.87 | 0.32 | 5.99 |
| **A3, 8-10cm** | 77.57 | 512 | 1327.65 | 98.54 | 0.17 | 5.35 |
| **A3, 10-12cm** | 66.22 | 704 | 2281.35 | 207.10 | 0.25 | 5.94 |
| **A3, 12-14cm** | 69.56 | 671 | 1798.61 | 239.31 | 0.30 | 5.93 |
| **A3, 14-16cm** | 83.11 | 413 | 919.00 | 55.75 | 0.12 | 4.91 |
| **A3, 16-18cm** | 87.65 | 277 | 863.90 | 22.59 | 0.07 | 3.99 |
| **A3, 18-20cm** | 87.78 | 293 | 797.64 | 28.14 | 0.09 | 4.22 |

**Supplementary Table 2.** Chemical data used to calculate thermodynamic-kinetic model. The DOC and SO_4_^2-^ data modified from Burton et al. (2011), sites A, C and E. Other chemical data used average values from Ward et al. (2014), site D2. The sulfide value used the theoretically highest amount 2μM, since its concentration was below the detection limit (Burton et al., 2011). Bicarbonate also used the highest concentration of 1.6mM, reported in the same study site (Johnston et al., 2011).

|  | DOC (mM) | | | SO_4_^2-^ (mM) | | |  | Other compounds | mg/L |
| --- | --- | --- | --- | --- | --- | --- | --- | --- | --- |
| Depth | A1 | A2 | A3 | A1 | A2 | A3 |  | Chloride | 19816.07 |
| 10 | 5 | 95 | 60 | 20 | 32 | 7 |  | Sodium | 11068.19 |
| 20 | 3 | 37 | 52 | 30 | 42 | 3 |  | Potassium | 402.38 |
| 30 | 1 | 32 | 32 | 30 | 41 | 6 |  | Calcium | 456.01 |
| 40 | 3 | 15 | 20 | 42 | 43 | 8 |  | Magnesium | 1203.65 |
| 50 | 5 | 13 | 19 | 42 | 42 | 8 |  | Phosphorus | 0.09 |
| 60 | 10 | 10 | 19 | 42 | 40 | 8 |  | Bromide | 84.63 |
| 70 | 9 | 10 | 19.5 | 42 | 40 | 8 |  | Barium | 25.90 |
| 80 | 8 | 10 | 10 | 41 | 44.5 | 10 |  | Aluminum | 0.01 |
| 90 | 6 | 10 | 4 | 40 | 44 | 17 |  | Nitrate | 0.02 |
| 100 | 4 | 10 | 0 | 39 | 45 | 18 |  | Nitrite | 0.004 |
| 110 | 3 | 7 | 0 | 39 | 43 | 19 |  | Orthophosphate | 0.003 |
| 120 | 2 | 3 | 0 | 39 | 37 | 19.5 |  | Ammonia | 0.05 |
| 130 | 4 | 0 | 0 | 38 | 35 | 20 |  |  |  |
| 140 | 0 | 0 | 0 | 37 | 34 | 20.3 |  |  |  |
| 150 | 0 | 0 | 0 | 36 | 32 | 20.4 |  |  |  |

**Supplementary Table 3.** Results generated from thermodynamic-kinetic model.

|  | ΔG_A_ | | | Thermodynamic factor | | | Kinetic factor | | |
| --- | --- | --- | --- | --- | --- | --- | --- | --- | --- |
| Depth | A1 | A2 | A3 | A1 | A2 | A3 | A1 | A2 | A3 |
| 10 | -101.20 | -110.16 | -110.22 | 0.80 | 0.89 | 0.84 | 0.96 | 0.97 | 0.88 |
| 20 | -101.65 | -108.85 | -110.54 | 0.79 | 0.87 | 0.81 | 0.97 | 0.98 | 0.77 |
| 30 | -99.94 | -108.50 | -109.29 | 0.75 | 0.87 | 0.82 | 0.97 | 0.98 | 0.87 |
| 40 | -103.36 | -107.46 | -108.25 | 0.81 | 0.85 | 0.81 | 0.98 | 0.98 | 0.90 |
| 50 | -105.18 | -107.11 | -108.07 | 0.82 | 0.85 | 0.81 | 0.98 | 0.98 | 0.90 |
| 60 | -107.34 | -106.46 | -107.95 | 0.84 | 0.84 | 0.81 | 0.98 | 0.98 | 0.90 |
| 70 | -107.47 | -106.46 | -108.01 | 0.84 | 0.84 | 0.81 | 0.98 | 0.98 | 0.90 |
| 80 | -107.51 | -106.40 | -106.62 | 0.83 | 0.84 | 0.80 | 0.98 | 0.98 | 0.92 |
| 90 | -107.09 | -106.34 | -104.33 | 0.83 | 0.84 | 0.78 | 0.98 | 0.98 | 0.95 |
| 100 | -106.35 | -106.27 | NA | 0.81 | 0.84 | NA | 0.98 | 0.98 | 0.00 |
| 110 | -105.87 | -105.39 | NA | 0.80 | 0.83 | NA | 0.98 | 0.98 | 0.00 |
| 120 | -105.08 | -103.30 | NA | 0.79 | 0.80 | NA | 0.97 | 0.97 | 0.00 |
| 130 | -107.00 | NA | NA | 0.81 | NA | NA | 0.98 | 0.00 | 0.00 |
| 140 | NA | NA | NA | NA | NA | NA | 0.00 | 0.00 | 0.00 |
| 150 | NA | NA | NA | NA | NA | NA | 0.00 | 0.00 | 0.00 |

## Supplementary Figures

**Supplementary Figure 1.** Diversity and richness values with 95% confidence intervals showed in error bars.

**Reference**

Burton, E. D., Bush, R. T., Johnston, S. G., and Sullivan, L. A. (2011). Sulfur biogeochemical cycling and novel Fe–S mineralization pathways in a tidally re-flooded wetland. *Geochimica et Cosmochimica Acta* 75, 3434–3451.

Johnston, S. G., Keene, A. F., Bush, R. T., Sullivan, L. A., and Wong, V. N. L. (2011). Tidally driven water column hydro-geochemistry in a remediating acidic wetland. *Journal of Hydrology* 409, 128–139.
